# Autophagy activation can partially rescue proteasome dysfunction‐mediated cardiac toxicity

**DOI:** 10.1111/acel.13715

**Published:** 2022-10-19

**Authors:** Eleni‐Dimitra Papanagnou, Sentiljana Gumeni, Aimilia D. Sklirou, Alexandra Rafeletou, Evangelos Terpos, Kleoniki Keklikoglou, Efstathios Kastritis, Kimon Stamatelopoulos, Gerasimos P. Sykiotis, Meletios A. Dimopoulos, Ioannis P. Trougakos

**Affiliations:** ^1^ Department of Cell Biology and Biophysics, Faculty of Biology National and Kapodistrian University of Athens Athens Greece; ^2^ Department of Clinical Therapeutics, School of Medicine National and Kapodistrian University of Athens Athens Greece; ^3^ Institute of Marine Biology, Biotechnology and Aquaculture, Hellenic Centre for Marine Research (HCMR) Crete Greece; ^4^ Biology Department University of Crete Heraklion Greece; ^5^ Service of Endocrinology, Diabetology and Metabolism Lausanne University Hospital and University of Lausanne Lausanne Switzerland

**Keywords:** autophagy, cardiotoxicity, metformin, mitostasis, proteasome inhibitor, proteostasis

## Abstract

The ubiquitin–proteasome pathway and its functional interplay with other proteostatic and/or mitostatic modules are crucial for cell viability, especially in post‐mitotic cells like cardiomyocytes, which are constantly exposed to proteotoxic, metabolic, and mechanical stress. Consistently, treatment of multiple myeloma patients with therapeutic proteasome inhibitors may induce cardiac failure; yet the effects promoted by heart‐targeted proteasome dysfunction are not completely understood. We report here that heart‐targeted proteasome knockdown in the fly experimental model results in increased proteome instability and defective mitostasis, leading to disrupted cardiac activity, systemic toxicity, and reduced longevity. These phenotypes were partially rescued by either heart targeted‐ or by dietary restriction‐mediated activation of autophagy. Supportively, activation of autophagy by Rapamycin or Metformin administration in flies treated with proteasome inhibitors reduced proteome instability, partially restored mitochondrial function, mitigated cardiotoxicity, and improved flies' longevity. These findings suggest that autophagic inducers represent a novel promising intervention against proteasome inhibitor‐induced cardiovascular complications.

## Graphical abstract

Heart‐targeted proteasome knockdown triggers proteotoxic stress and loss of mitostasis causing cardiotoxicity and systemic effects. Autophagy activation in the heart rescues proteasome knockdown‐mediated cardiotoxicity. Metformin activates autophagy and alleviates proteasome inhibitors' cardiotoxicity.

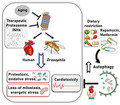

**Abbreviations**
ALP: autophagy lysosome pathway
Atg: autophagy‐related gene
BTZ: bortezomib
CFZ: carfilzomib
C‐L/LLE: caspase‐like peptidase activity
CT‐L/LLVY: chymotrypsin‐like peptidase activity
ER: endoplasmic reticulum
MET: metformin
PI: proteasome inhibitor
PN: proteostasis network
PR: protein restriction
RAP: rapamycin
ROS: reactive oxygen species
SQSTM1: sequestosome 1
UPP: ubiquitin proteasome pathway

## 1 INTRODUCTION

Protein quality control maintains proteome homeodynamics (proteostasis) and is critical for cellular functionality and viability. Cell proteostasis is maintained by the action of a wired highly integrated compartment‐specific system, namely the proteostasis network (PN) (Labbadia & Morimoto, 2015). Major components of the PN are the protein synthesis and sorting/trafficking machineries, the endoplasmic reticulum (ER) unfolded protein response (UPR^ER^), the molecular chaperones, and the two main degradation machineries, namely the autophagy‐lysosome (ALP) and the ubiquitin–proteasome (UPP) pathways (Pohl & Dikic, 2019).

The autophagy‐lysosome is a conserved degradation process that includes microautophagy, chaperone‐mediated autophagy, and macroautophagy (Klionsky et al., 2016). Macroautophagy involves the formation of double‐membrane vesicles (autophagosomes), which, by the participation of autophagy‐related (Atg) proteins, sequester cytoplasmic portions, damaged polypeptides or organelles and transfer them to lysosome for degradation. Atg8 (along with its lipidated form) is a major driver for autophagosome maturation, and in mammals, the Atg8 family consists of six members divided into the LC3 and GABARAP subfamilies (Schaaf et al., 2016). ALP can also degrade ubiquitinated polypeptides and protein aggregates or ubiquitin decorated organelles (e.g., mitochondria) via the direct binding of SQSTM1 (sequestosome 1, also known as p62) to ubiquitinated substrates (Klionsky et al., 2016). On the contrary, UPP degrades normal short‐lived ubiquitinated proteins during their physiological recycling and non‐repairable unfolded or misfolded polypeptides (Tsakiri & Trougakos, 2015). The 26S eukaryotic proteasome is a complicated protein machine that comprises a 20S core particle (CP) bound to 19S regulatory particles (RP). The 20S CP consists of four stacked heptameric rings (two *α* surrounding two of *β* type) that form a barrel‐like structure; the caspase (C‐L), trypsin (T‐L), and chymotrypsin (CT‐L) like peptidase activities are located at the beta 1, beta 2, and beta 5 (known as Prosβ1, Prosβ2, and Prosβ5 in *Drosophila*) proteasomal subunits, respectively (Livneh et al., 2016).

As we and others have shown, loss of proteostasis including declined proteasome activity are major hallmarks of aging (López‐Otín et al., 2013; Tsakiri & Trougakos, 2015). On the contrary, aberrant proteasome activation is found in advanced tumors (Sklirou et al., 2018), and thus, proteasome inhibition provides a promising novel anti‐tumor therapy (Manasanch & Orlowski, 2017). Consistently, several selective proteasome inhibitors (PIs), including Bortezomib (BTZ, a slowly reversible PI) and Carfilzomib (CFZ, binds irreversibly to proteasome), have demonstrated clinical efficacy in the treatment of hematologic malignancies, for example, multiple myeloma (MM), and are being evaluated for the treatment of other types of cancer (Dimopoulos et al., 2015). Nonetheless, and despite the fact that therapeutic PIs have revolutionized MM treatment, the emergence of severe adverse effects (AEs) such as peripheral neuropathy and/or cardiotoxicity remain a significant limitation in the clinic (Cornell et al., 2019; Dimopoulos et al., 2016; Kastritis et al., 2021). Cardiomyocytes are terminally differentiated post‐mitotic cells exhibiting very limited regenerative capacity; also, they are constantly exposed to proteotoxic, metabolic, and mechanical stress and are thus susceptible to reduced proteasome functionality (Patterson et al., 2007). Hence, therapeutic PI‐mediated proteasome dysfunction is likely a causative factor of cardiac malfunction in MM patients (Hasinoff et al., 2017). In support, cardiomyocyte‐restricted genetic inhibition of proteasome CT‐L activity in a mouse model resulted in increased (vs. controls) cardiomyocyte apoptosis and ischemia/reperfusion injury (Tian et al., 2012), as well as in cardiac malfunction during systolic overload (Ranek et al., 2015); proteasome malfunction in this model induced myocardial macroautophagy via the calcineurin‐TFEB‐p62/SQSTM1 pathway (Pan et al., 2020). Despite these interesting findings, the mechanistic details and downstream effects of heart‐targeted proteasome loss of function remain poorly understood at in vivo experimental settings.

By exploiting the *Drosophila* in vivo model, we recently mapped the extensive functional crosstalk of proteostatic and mitostatic modules (Gumeni et al., 2019; Tsakiri, Gumeni, Iliaki, et al., 2019; Tsakiri, Gumeni, Vougas, et al., 2019). Also, our preliminary analyses showed that systemic administration of either CFZ or BTZ in young flies led to disruption of proteostasis and caused perturbation of cardiac functionality (Tsakiri et al., 2017). *Drosophila* is particularly suitable for studying cardiac AEs of therapeutic PIs before moving to the far more complex and time‐consuming mammalian models, due to its powerful genetics, the fact that flies' proteasomes structurally resemble those of mammals (Tsakiri & Trougakos, 2015), and also because the fruit fly is the only major invertebrate model organism that contains a beating heart tube and a circulatory system with developmental and functional homologies to the vertebrate heart (Piazza & Wessells, 2011). Moreover, the fly and mammalian heart/cardiomyocytes share many similar traits in conditions of declined proteostasis (e.g., during aging), including systolic and diastolic dysfunction, increased arrhythmia, and decreased metabolic fitness (Blice‐Baum et al., 2019). In support, as we showed recently, findings in the fly model (Gumeni et al., 2019; Tsakiri et al., 2017; Tsakiri, Gumeni, Iliaki, et al., 2019; Tsakiri, Gumeni, Vougas, et al., 2019) can be readily translated in mice (Efentakis et al., 2019) and in informative clinical studies (Kastritis et al., 2021; Papanagnou et al., 2018). We report here that heart‐specific partial loss of proteasome activity results in increased proteotoxic and energetic stress in the heart, leading to disrupted cardiac functionality, systemic toxicity, and reduced longevity. These phenotypes can be partially rescued by heart targeted or by systemic pharmacological activation of autophagy.

## 2 RESULTS

### 2.1 Targeted proteasome dysfunction in *Drosophila* heart disrupts cardiac activity, causes systemic effects, and accelerates aging

Initially, we investigated the age‐related effects on proteasome functionality specifically in the fly heart. We found a significant reduction of the rate limiting for protein breakdown CT‐L proteasomal activity in isolated fly hearts from aged (52–55 days old) vs. young (7–10 days old) flies (Figure S1A). Furthermore, staining and immunoblotting analysis of isolated heart tissues for ref(2)P (the fly ortholog of mammalian SQSTM1/p62, a well‐characterized autophagosome substrate) expression revealed its accumulation in aged hearts (Figure S1B), possibly suggesting increased proteome instability (Lim et al., 2015; Tsakiri, Gumeni, Vougas, et al., 2019) and reduced autophagic flux (Klionsky et al., 2016). Furthermore, LysoTracker tissue staining and lysosomal associated protein 1 (Lamp1, lysosomal marker) expression analysis showed a significant reduction of lysosomes and Lamp1 expression (Figure S1C), along with a moderate decrease of cathepsins activity (Figure S1D), in aged flies heart tissue. Also, aged flies displayed a deterioration of cardiac functionality, being evident by bradycardia, that is, reduced number of heart beats (Figure S1E,F).

**FIGURE 1:**
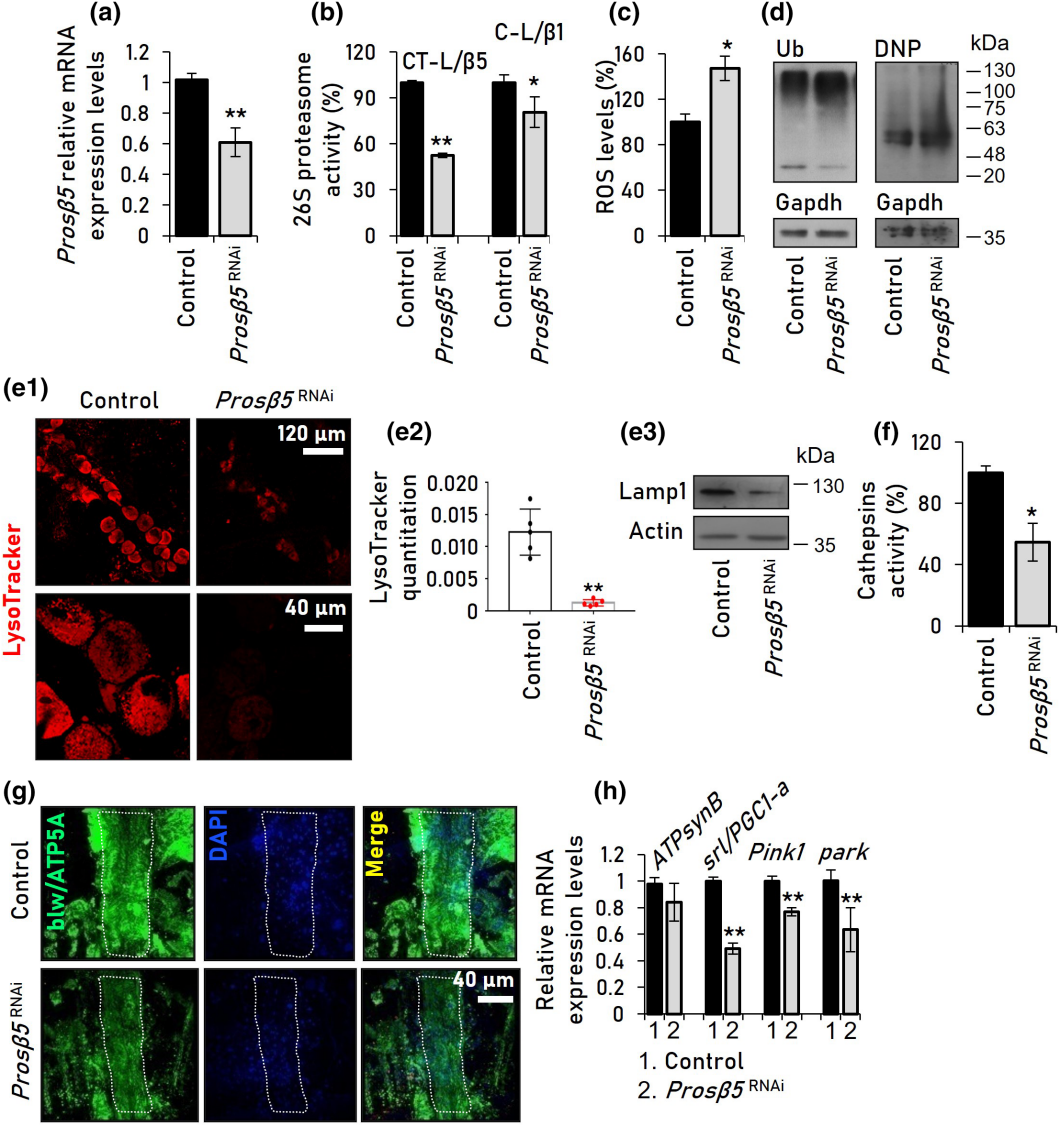
Heart‐targeted (Gal4^ΤinCΔ4^) KD of the proteasomal *Prosβ5* gene results in proteome instability and reduced mitochondria number. (a) Relative *Prosβ5* gene expression (vs. control) in heart tissues following *Prosβ5* siRNA. (b, c) Relative (%) 26S proteasome activities (b) and ROS levels (c) in heart tissues of *Prosβ5* ^RNAi^ (vs. control) flies. (d) Immunoblot analyses of proteome ubiquitination (Ub) and carbonylation (DNP) in flies' heart tissues after *Prosβ5* KD. (e) CLSM viewing of *Prosβ5* ^RNAi^ (vs. control) flies heart tubes stained with LysoTracker (e1), LysoTracker quantitation (e2), and immunoblotting analysis using the lysosomal marker anti‐Lamp1 (e3). (f) Relative (%) cathepsins activity in heart tissues of flies with the shown genotypes. (g) CLSM visualization of mitochondria in heart tissues of the shown fly lines after blw/ATP5A immunofluorescence staining; nuclei were counterstained with DAPI. (h) Relative expression levels (vs. control) of indicated mitochondrial genes in isolated heart tissues of the shown genotypes following *Prosβ5* KD. In (a, h) gene expression was plotted vs. respective controls; *RpL32/rp49* gene was used as RNA input reference. Gapdh and Actin probing in (d) and (e3), respectively, were used as protein input reference. *p* Values were calculated with unpaired *t* test. Bars, ±SD (*n* ≥ 3); *p < 0.05; **p < 0.01

To study the effects of unbalanced proteostasis on heart functionality, we then induced siRNA‐mediated knockdown (KD) of the *Prosβ5* proteasomal gene (Figure 1a) (CT‐L activity) in the cardiac tissue [Gal4^ΤinCΔ4^ driver (Lo & Frasch, 2001)] of young flies. We found that *Prosβ5* KD suppressed mostly the targeted CT‐L (but also the C‐L) proteasome activity in the heart tissue (Figure 1b), increased ROS levels (Figure 1c), and induced the accumulation of ubiquitinated and carbonylated proteins (Figure 1d). Moreover, it reduced lysosomes number and Lamp1 expression (Figure 1e), and it also suppressed cathepsins activity (Figure 1f). Heart‐targeted *Prosβ5* KD also led to decreased mitochondria number in young flies' heart tube (Figure 1g); this readout was combined with downregulation of mitophagy‐related genes (*Pink1, park*) and of the mitochondrial biogenesis master regulator gene *srl* [(spargel) also known as *PGC1‐a*, the fly ortholog of mammalian *PPARGC1A*; peroxisome proliferator‐activated receptor gamma coactivator 1‐alpha] (Figure 1h). Thus, proteasome dysfunction in young flies' heart induces extended collapse of proteostatic and mitostatic modules causing (among others) proteome instability.

**FIGURE 2:**
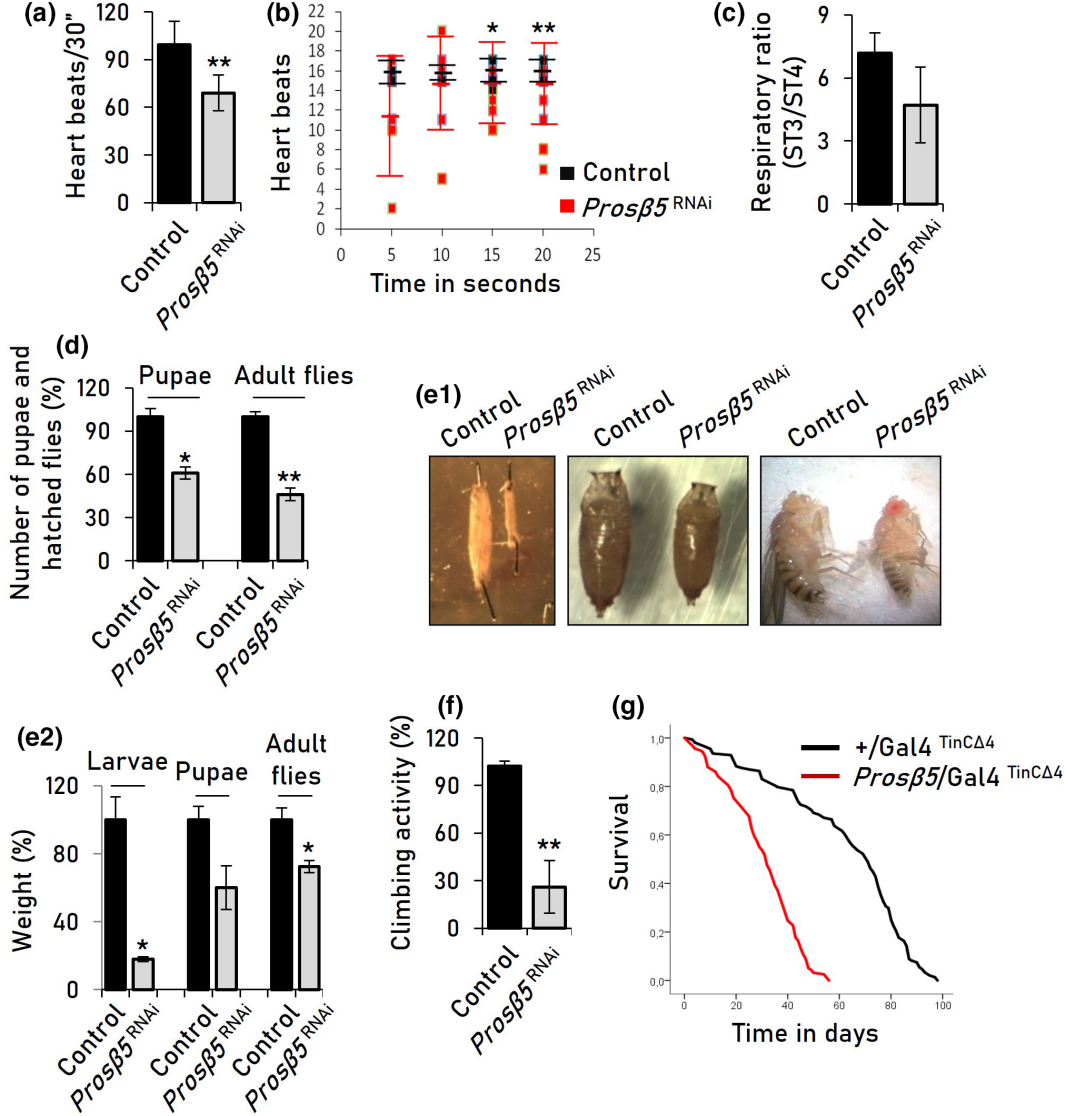
Heart‐specific (Gal4^ΤinCΔ4^) *Prosβ5* KD causes cardiotoxicity, developmental disorders, and acceleration of aging‐related phenotypes. (a) Heart beats (mean from 15 female adult flies) normalized to a 30 sec period (see, Videos S1 and S2). (b) Heart rhythm calculated by beats per 5 sec; each dot represents the number of heart beats/5 sec. (c) Mitochondrial ST3/ST4 respiratory ratio in somatic tissues of the indicated genotypes. (d) Number (%) of pupae and hatched flies (at 7 and 14 days, respectively) after transferring 30 embryos per assay of the shown genotypes to culture medium. (e) Representative images (e1) and weight (%) (e2) of larvae, pupae, and female adults of control and *Prosβ5* ^RNAi^ flies. (f) Climbing activity (%) of young transgenic flies of the indicated genotypes. (g) Longevity curves of control flies or after *Prosβ5* gene KD; log‐rank, Mantel‐Cox test: Control vs. *Prosβ5* ^RNAi^ *p* < 0.0001. Statistics of the longevity curves are also reported in Table S1. *p* values in (a–f) were calculated with unpaired *t* test. Bars, ±SD (*n* ≥ 3); *p < 0.05; **p < 0.01

Cardiac dysfunction in young flies due to targeted loss of proteasome functionality was manifested by reduced number of heart beats (bradycardia) (Figure 2a) and arrhythmia (arrhythmic heart rate) (Figure 2b, Videos S1 and S2). Specifically, although control flies presented a relatively stable number of beats ranging from 3 to 3.6 beats/sec, proteasome dysfunction in the heart resulted in significant irregularity with 0.2–4 beats/sec (Figure 2b). Interestingly, despite non‐significant inhibition of the CT‐L proteasome activity in other than cardiac tissues after *Prosβ5* KD (Figure S2A), we found that heart‐specific proteasome KD tended to trigger mitochondrial respiratory deficiency at the whole organism level (Figure 2c), indicating systemic energetic stress. The systemic effects were also evident by disrupted developmental processes, since *Prosβ5* KD in the heart significantly reduced pupation and flies' hatching rates (Figure 2d). Also, heart‐specific *Prosβ5* KD is associated with reduced size of both larvae and adults (Figure 2e), a phenotype indicative of severe systemic metabolic stress. Finally, heart‐targeted proteasome KD accelerated age‐related phenotypes, as it caused early defective locomotion (Figure 2f) and reduced longevity (Figure 2g, Table S1). Similar effects, albeit less intense, were found after *Prosβ1* (C‐L activity) KD (not shown); all reported phenotypes were specific to *Prosβ5* KD as none was evident in a *mCherry* ^RNAi^ transgenic line (not shown).

Taken together, these findings highlight the cardiac tissue dependence on proper proteasome functionality; also, they suggest that heart‐specific disruption of proteasome function in young flies induces systemic effects and accelerates aging.

### 2.2 Heart‐targeted autophagy activation alleviated *Prosβ5* KD‐mediated toxicity and partially rescued cardiac function

The severe impact of proteasome dysfunction due to reduced CT‐L activity on heart functionality could also explain the cardiotoxicity seen in the clinic by the CT‐L‐specific therapeutic PIs (e.g., CFZ) used for MM treatment (Dimopoulos et al., 2016; Kastritis et al., 2021). In line with our previous findings showing enhanced ubiquitination of dysfunctional proteasomes (Tsakiri et al., 2013), we found increased co‐localization of proteasomes with Atg8a (Figure S2B1) in dissected hearts expressing *Prosβ5* RNAi; this observation indicates increased targeting of unstructured and/or dysfunctional proteasomes to proteaphagy (data to be presented elsewhere). We also observed the upregulation of other proteasome proteins, that is, 26Sα (20S complex) and P54/Rpn10 (19S complex) levels after *Prosβ5* KD, suggesting a possible feedback loop mechanism aiming to restore normal proteasome functionality (Figure S2B).

Interestingly, treatment of isolated semi‐intact hearts after *Prosβ5* KD with bafilomycin A1 (BAF; Chang et al., 2020) revealed no further accumulation of the lipidated Atg8a/GABARAP form suggesting (in this experimental setting) a likely block of autophagic flux (Figure S3A). In support to reduced autophagic flux, we found that *Prosβ5* KD resulted in accumulating ref(2)P‐GFP [not amenable to transcriptional regulation (Klionsky et al., 2016)] (Figure S3B,C) and Atg8a [Figure S3B; as reported (Jacomin et al., 2020), Atg8a also showed nuclear localization] in larvae muscle tissue, as well as, that *Prosβ5* ^RNAi^ reduced GFP‐Lamp1 and lysotracker co‐localization in larvae heart tissues (Figure S3D), indicating loss of lysosomes acidification (Johnson et al., 2016). Thus, prolonged *Prosβ5* KD also suppresses the autophagic machinery.

**FIGURE 3:**
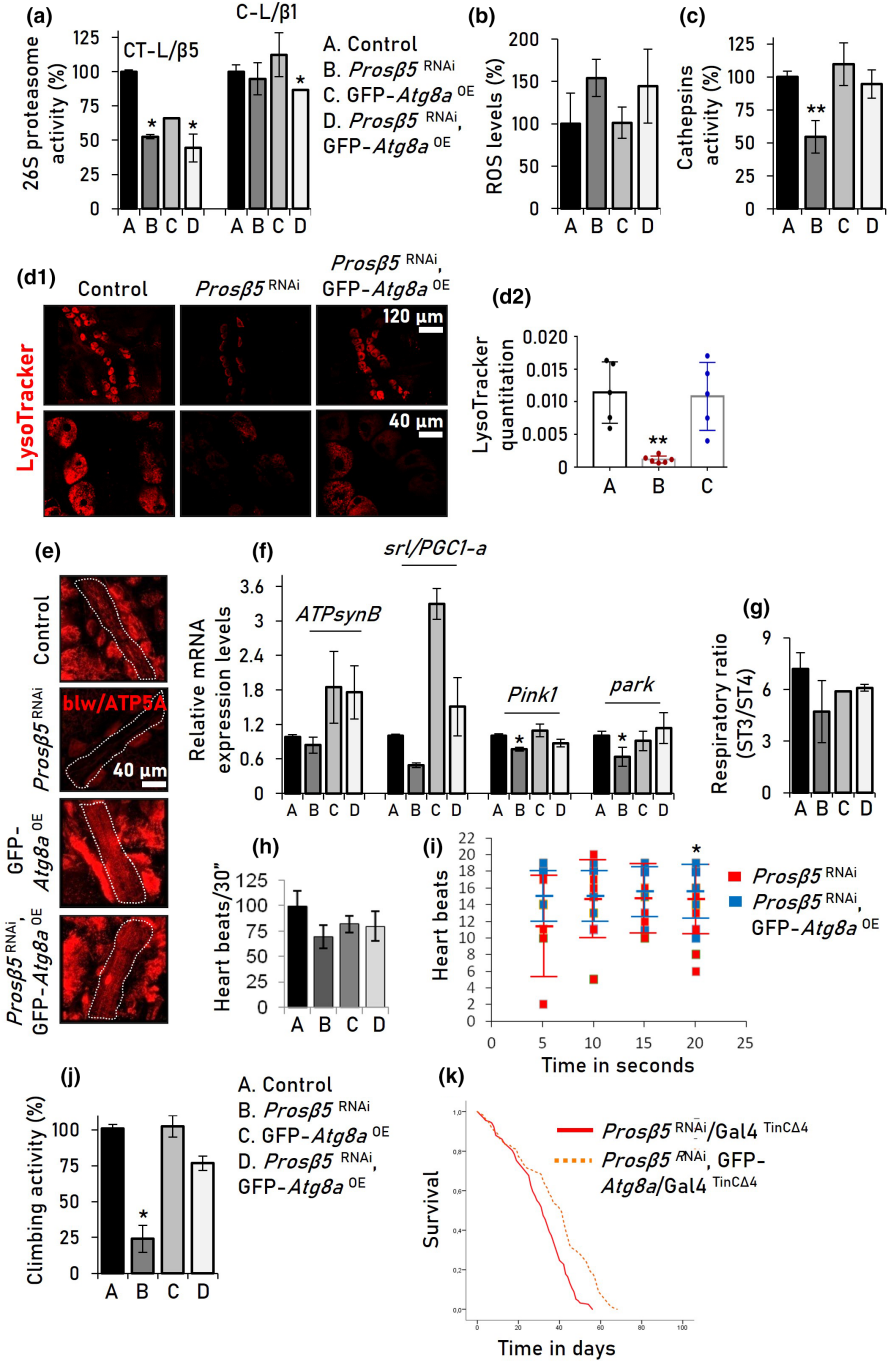
Heart‐targeted (Gal4^ΤinCΔ4^) *Atg8a* ^OE^ in adult flies suppresses the toxic effects of proteasome KD and partially rescues mitochondria and cardiac functionality. (a–c) Relative (%) 26S proteasome activities (a), ROS levels (b), and cathepsins activity (c) in the heart tissues of the indicated transgenic lines. (d) CLSM visualization of shown fly lines heart tubes following LysoTracker staining (d1) and LysoTracker quantitation (d2). (e) CLSM visualization of mitochondria following blw/ATP5A immunofluorescence staining of transgenic flies' heart tissues. (f) Relative expression levels of mitochondrial genes in heart tissues of the indicated transgenic flies. (g) Mitochondrial ST3/ST4 respiratory efficiency rates from somatic tissues of flies expressing the shown transgenes, specifically in heart. (h) Heart beats (mean from 15 female adult flies) normalized to a 30 sec period (see, Videos S1 and S3). (i) Heart rhythm calculated by beats per 5 sec; each dot represents the number of heart beats/5 sec. (j) Climbing activity (%) of young transgenic flies vs. control. (k) Longevity curves of the indicated transgenic lines; log‐rank, Mantel‐Cox test: *Prosβ5* ^RNAi^ vs. *Prosβ5* ^RNAi^, GFP‐*Atg8a* ^ΟΕ^ *p* < 0.001. Statistics of the longevity curves are also reported in Table S1. Gene expression in (f) was plotted vs. respective controls; *RpL32/rp49* gene expression was used as RNA input reference. *p* Values were calculated with one‐way ANOVA with Kruskal–Wallis test in (a–d, f–h, j) and with unpaired *t* test in (i). Bars, ±SD (*n* ≥ 3); *p < 0.05; **p < 0.01

We thus initially investigated whether the toxic effects of CT‐L inhibition in the heart and systemically can be alleviated by genetically activating autophagy. To this end, we overexpressed *Atg8a* in the *Prosβ5* ^RNAi^ background [this intervention did not affect *Prosβ5* downregulation (Figure S4A)], and found that, although *Atg8a* upregulation did not increase proteasome activity in the cardiac tissues (Figure 3a) and it did not suppress ROS accumulation (Figure 3b), it restored normal cathepsins activity (Figure 3c) and enhanced lysosomes number (Figure 3d). Consistently to cathepsins increased activity, which is indicative of activated autophagy (Bullón et al., 2018; Tatti et al., 2013; Xu et al., 2021), Atg8a overexpression (OE) in the *Prosβ5* ^RNAi^ background seemingly enhanced autophagic flux in flies' heart and whole body (Figure S4B) and decreased ubiquitinated and carbonylated proteins (Figure S4C). Also, it normalized mitochondrial number as compared to *Prosβ5* ^RNAi^ flies (Figure 3e), upregulated mitostatic genes expression, including the mitochondrial biogenesis regulator *srl/PGC1‐a*, (Figure 3f), and tended to improve mitochondria respiration rates (Figure 3g). Additionally, *Atg8a* ^OE^ in the *Prosβ5* ^RNAi^ background tended to increase heart beats and restored a more regular and stable heart rhythm (number of beats/sec) (Figure 3h,i, Videos S1–S3), improving thus cardiac function. Further, heart‐specific *Atg8a* ^OE^ mitigated *Prosβ5* KD‐mediated systemic developmental defects and growth retardation (Figure S4D); it also tended to restore physiological thickness and dimensions of the heart tube's conical chamber (Figure S4E) and increased *Prosβ5* ^RNAi^ flies' neuromuscular (locomotion) activity and lifespan (Figure 3j,k, Table S1).

Hence, the toxic effects of proteasome dysfunction in the heart can be partially rescued by heart‐targeted *Atg8a* ^OE^, underlying the protective role of ALP upon proteasome dysfunction.

### 2.3 Systemic protein restriction (PR) attenuates heart‐targeted proteasome KD‐mediated toxicity

Since caloric restriction (CR) promotes autophagy (Bagherniya et al., 2018), we subjected *Prosβ5* ^RNAi^ flies to low protein intake, which has demonstrated healthspan/lifespan benefits in a variety of model organisms (Mirzaei et al., 2016). We selected PR (reduced amino acid and protein availability) for this intervention since several studies have suggested that it contributes to about half of the lifespan extension mediated by CR (Pamplona & Barja, 2006). By using an Atg8a/GABARAP antibody for staining the fly Atg8a protein (Chang et al., 2020) in +/GFP*‐Lamp1*, Gal4^TinCΔ4^ flies, we found that PR augmented Atg8a‐Lamp1 co‐localization, indicating increased fusion of lysosomes with autophagosomes and hence enhancement of autophagy flux in heart tissues after PR (Figure S5A1); PR also resulted in marginal p‐Ampka and significant foxo accumulation in flies' somatic tissues (Figure S5A2).

Furthermore, *Prosβ5* ^RNAi^ (or *Prosβ1* ^RNAi^; not shown) flies fed with low protein medium displayed (vs. controls) increased mitochondria number in the heart tissue (Figure S5B) and improved heart functionality (Figure S5C, Videos S2 and S4). Moreover, low protein intake partially suppressed heart‐targeted proteasome KD‐mediated acceleration of aging (Figure S5D, Table S1), further supporting the beneficial effects of autophagy activation via systemic dietary restriction on heart‐targeted proteasome dysfunction‐mediated toxicity.

### 2.4 Pharmacological activation of autophagy mitigates the toxic effects caused by administration of PIs

Given the cardiotoxicity of therapeutic PIs in both the clinic (Cornell et al., 2019) and the fly model (Tsakiri et al., 2017), we then asked whether pharmacological systemic activation of autophagy could ameliorate this severe AE. We thus exposed wild‐type flies to BTZ (1 μM) or CFZ (50 μM) for 7 days and combined (or not) the treatment with administration of the autophagy inducer RAP (100 μM); at the used concentrations, BTZ and CFZ inhibit the proteasomal CT‐L activity by ~20%–30% (Tsakiri et al., 2017). We found that RAP did not affect the PI‐mediated proteasome inhibition (Figure S6A). It also increased (vs. flies exposed solely to PIs) mitochondrial number in the cardiac tube (Figure S6B) and restored a more physiological heart function (Figure S6C,D).

**FIGURE 4:**
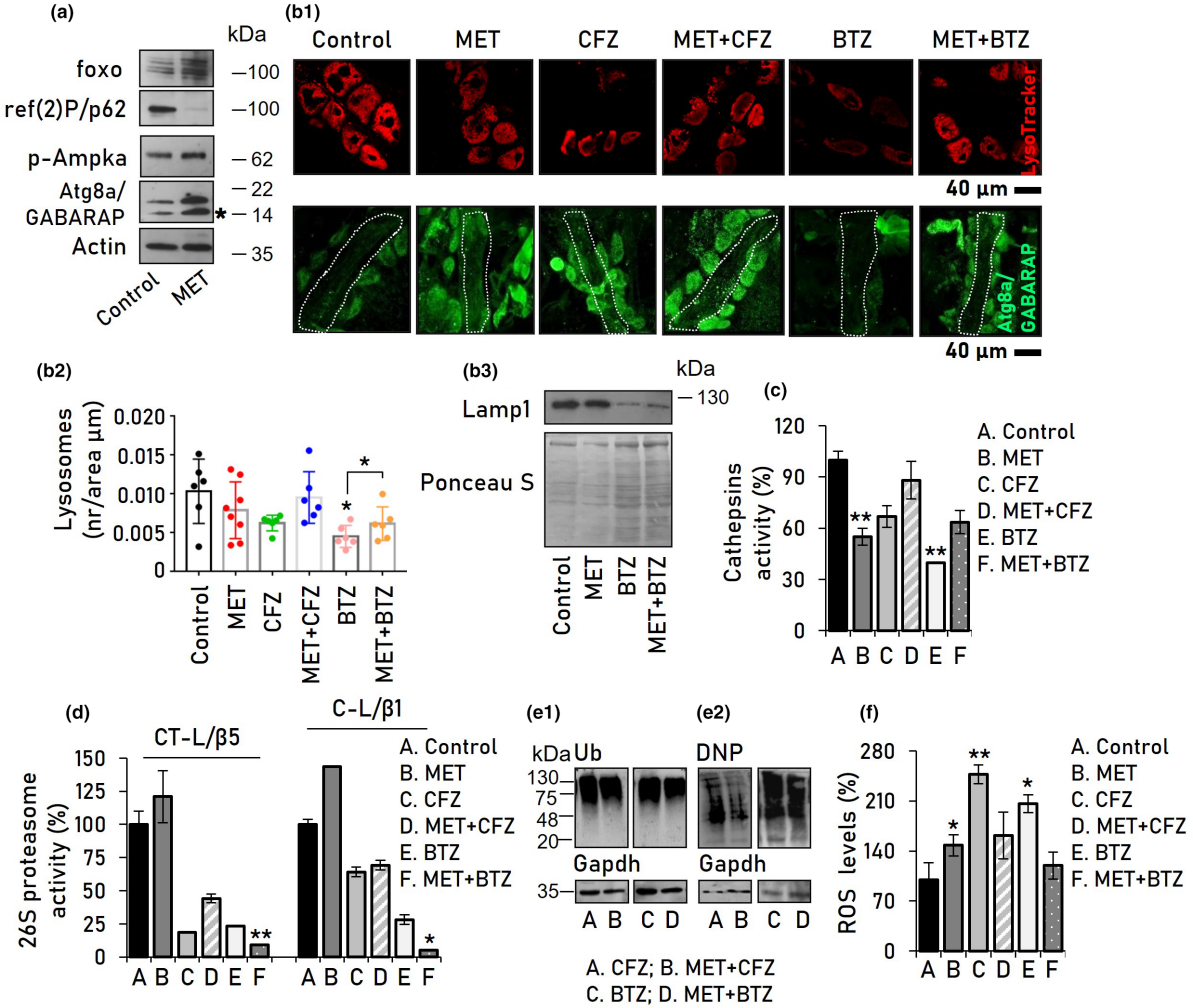
MET treatment promotes autophagy and reduces proteotoxic stress caused by PIs administration. (a) Immunoblot analyses of dissected flies' hearts after treatment with MET; samples were probed with antibodies against foxo, ref(2)P/p62, p‐Ampka, and Atg8a/GABARAP. (b) CLSM visualization of flies' heart tubes following LysoTracker and immunofluorescence Atg8a/GABARAP staining (b1) along with quantitation of lysosomes number (b2) and immunoblotting analysis using the lysosomal marker anti‐Lamp1 (b3). (c) Relative (%) cathepsins activity in heart tissues of control flies and flies exposed to MET and/or CFZ, BTZ. (d) Relative (%) 26S proteasome activities in heart tissues of flies after treatment with the indicated drugs. (e) Immunoblot analyses of total protein ubiquitination (Ub) (e1) or carbonylation (DNP) (e2) in heart tissues of flies treated with the shown drugs. (f) Relative (%) ROS levels in heart tissues following treatment of flies with the indicated drugs. Concentrations of used drugs were as follows: MET (1 mM), CFZ (50 μM), and BTZ (1 μM). Flies were treated with the indicated drugs for 14 days. Asterisk (*) in (a) indicates the lipidated Atg8a form. Actin probing in (a), ponceau S staining in (b3), and Gapdh probing in (e) were used as reference for protein input. *p* Values were calculated with one‐way ANOVA with Kruskal–Wallis test. Bars, ±SD (*n* ≥ 3); *p < 0.05; **p < 0.01

We then examined the anti‐glycemic MET (an FDA and EMA‐approved drug), which is also considered as an autophagy activator (Kulkarni et al., 2020). After screening a broad range of MET concentrations in young flies, we selected the concentration of 1 mM for our studies, as this concentration upregulated foxo, p‐Ampka, and Atg8a‐I (unlipidated form), Atg8a‐II (lipidated form) protein expression levels, parallel to ref(2)P/p62 downregulation in flies' hearts (Figure 4a). Immunofluorescence staining of Atg8a/GABARAP in flies expressing GFP‐*Lamp1* and treated with MET showed that MET led to increased autophagosome and lysosome co‐localization (Figure S7A). Furthermore, staining with LysoTracker and an Atg8a/GABARAP antibody revealed that co‐administration of MET with either BTZ or CFZ, increased (vs. solely BTZ or CFZ treatment) lysosome and autophagosome numbers in the heart tissue of treated flies (Figure 4b). It also largely normalized cathepsins activity in heart tubes of CFZ or BTZ‐treated flies (Figure 4c), suggesting enhanced autophagic flux (Xu et al., 2021). Consistently, treatment of flies with 1 mM MET decreased BTZ‐ or CFZ‐mediated ref(2)P/p62 and Atg8a accumulation in larvae and adult flies' tissues (Figure S7B,C) indicating enhanced autophagic flux (Klionsky et al., 2016). Co‐administration of MET did not increase proteasome activities in PI‐treated flies' heart tissue (Figure 4d). Yet, it mitigated proteotoxic and oxidative stress, especially in combination with CFZ, as was evident by reduced accumulation of carbonylated (but not ubiquitinated) proteins (Figure 4e) and tended to suppress PI‐mediated redox imbalance (Figure 4f).

**FIGURE 5:**
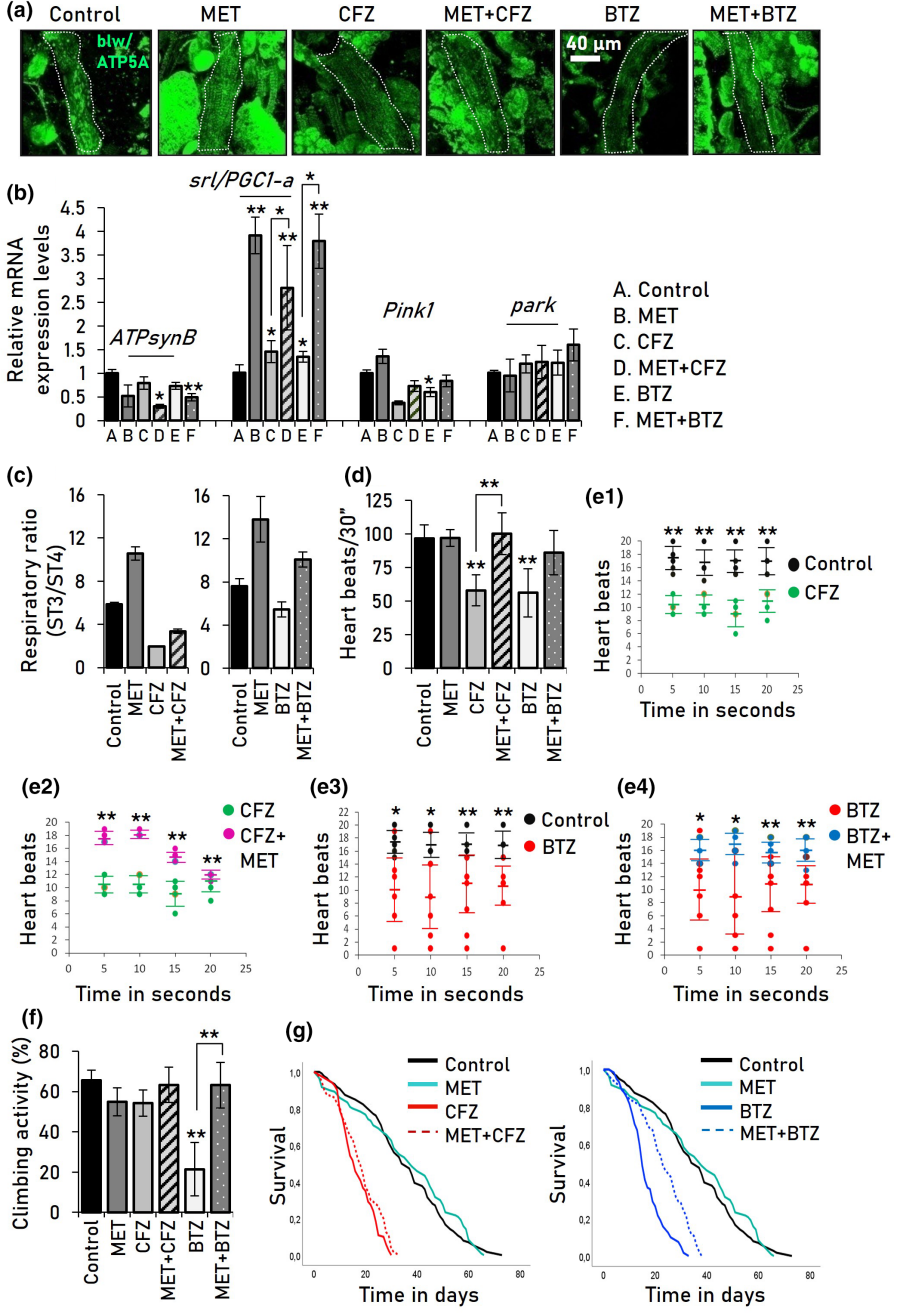
MET induces mitochondrial biogenesis and restores mitochondria and cardiac functionality when co‐administrated with PIs. (a) CLSM visualization of heart tissues mitochondria following blw/ATP5A immunofluorescence staining. (b) Relative expression levels of shown mitochondrial genes following treatment (or not) of flies with the indicated drugs. (c) Mitochondrial ST3/ST4 respiratory efficiency rates (vs. control) from somatic tissues of flies exposed to shown drugs for 14 days. (d) Heart beats (mean from 20 female adult flies) normalized to a 30 sec period (see, Videos S5 and S6). (e) Heart rhythm calculated by beats per 5 sec; each dot represents the number of heart beats/5 sec. (f) Locomotion (climbing) activity (%) and (g) longevity curves of flies exposed (or not) to the shown drugs; log‐rank, Mantel‐Cox test: Control vs. MET *p* = 0.5, control vs. CFZ *p* < 0.0001, CFZ vs. ΜET + CFZ *p* = 0.03, control vs. BTZ *p* < 0.0001, BTZ vs. ΜET + BTZ *p* < 0.0001. Statistics of the longevity curves are also reported in Table S1. Concentrations of used drugs were as follows: (MET 1 mM), CFZ (50 μM), and BTZ (1 μM). Gene expression in (b) was plotted vs. respective controls. *RpL32/rp49* gene expression in b was used as input reference. *p* Values were calculated in (b–d, f) with one‐way ANOVA with Kruskal–Wallis test and in (e) with unpaired *t* test. Bars, ±SD (*n* ≥ 3); *p < 0.05; **p < 0.01

Furthermore, we found that MET administration increased (vs. solely CFZ or BTZ‐treated flies) mitochondria number (Figure 5a) in PI‐treated flies' heart tube; it also augmented the expression level of the *srl/PGC1‐a* gene (Figure 5b) and mitochondria respiratory state (ST3:ST4) (Figure 5c) of PI‐treated flies, suggesting that MET treatment partially normalized tissue energetics. In support, although lipid levels were not further reduced (vs. solely MET or PIs treatment) in MET and CFZ or BTZ co‐treated flies' fat body (Figure S8A), exposure to MET tended to normalize (mostly vs. solely CFZ‐treated flies) the expression levels of the triglyceride lipase *Atgl/bmm* and of the lipid droplets regulating *Lsd‐1*, *Lsd‐2* (Figure S8B) genes, indicating metabolic rebalancing.

MET also restored a more physiological heart functionality, as it largely normalized flies' heart beats (Figure 5d, Videos S5 and S6) and decreased arrhythmia events presented after CFZ or BTZ administration (Figure 5e). Finally, MET co‐administration suppressed the PI‐related pro‐aging phenotypes, as it was found to improve flies' locomotion activity (Figure 5f) and lifespan/healthspan of young (Figure 5g, Table S1) and middle‐aged (not shown) flies.

Overall, pharmacological inducers of autophagy (e.g., RAP or MET) can alleviate therapeutic PI‐mediated cardiotoxicity.

## 3 DISCUSSION

Proteasome is central to maintenance of PN functionality, representing a key regulator of cell growth and survival in eukaryotic cells. Whereas the decline of its activity during aging (particularly in post‐mitotic tissues) contributes to age‐related phenotypes and degenerative diseases (Kaushik & Cuervo, 2015; Tsakiri & Trougakos, 2015), certain tumors become addicted to high proteasome activities likely due to excessive proteome instability and oxidative damage (Sklirou et al., 2018). Consistently, although PIs have revolutionized the therapy of hematologic malignancies (Dimopoulos et al., 2015), their use in the clinic is marked by severe AEs, such as peripheral neuropathies and/or heart failure (Dimopoulos et al., 2016). Neurons and cardiomyocytes share common features, as they are terminally differentiated cells, characterized by limited ability to dilute accumulating proteome damage (Rujano et al., 2006). Particularly, cardiomyocytes are highly specialized cells with elevated metabolic demands and constant exposure to increased proteotoxic, oxidative, and mechanical stress; therefore, they are highly dependent on proper PN functionality (Fan et al., 2020; Gupta & Robbins, 2016). In support, we found that heart‐targeted proteasome KD in young flies correlated with heart failure, phenocopying the cardiovascular complications seen in the clinic by therapeutic PIs (Cornell et al., 2019).

Similarly to either genetic‐ or PI‐mediated proteasome dysfunction at the whole‐animal level (Tsakiri, Gumeni, Vougas, et al., 2019); targeted proteasome KD in flies' cardiac tissues triggered oxidative, proteotoxic, and energetic stress. Reportedly, proteasome disruption has been shown to trigger the activation of autophagy (Pan et al., 2020); yet, in line with previous findings in tumor cells (Kao et al., 2014) or in fly myocytes (Zirin et al., 2015), we observed that sustained targeted proteasome inhibition in cardiac tissues disrupted autophagy, decreased lysosomal number, and disrupted pH in the lysosomal lumen. Given that lysosomes generate and maintain their pH gradients, by using the activity of a proton‐pumping V‐type ATPase, which uses metabolic energy in the form of ATP to pump protons into the lysosome lumen (Ishida et al., 2013; Todkar et al., 2017), our findings indicate a rather generalized collapse of proteostatic machineries, probably due to impaired cellular energetics. Notably, proteasome malfunction resulted in significantly decreased mitochondrial number consistent with studies showing that loss of mitostasis and PRKN‐mediated ubiquitination of the outer mitochondrial membrane proteins recruits SQSTM1/p62 to mitochondria, where it is thought to promote mitophagy via its capacity to directly interact with the MAP1LC3/LC3 (Tanaka et al., 2010). Mitochondria maintain cellular energetics through oxidative phosphorylation, while dysfunctional mitochondria increase ROS production and even promote cell death upon excessive cell damage (Giacomello et al., 2020). Given the mitochondrial evolution and the physical isolation of their contents, regulation of mitostasis was thought to be independent of UPP. Nonetheless, it was shown that numerous mitochondrial proteins are subjected to ubiquitination (Jeon et al., 2007; Peng et al., 2003), as well as that UPP mediates the degradation of inter‐ and outer‐membrane (Kowalski et al., 2018; Metzger et al., 2020) mitochondrial proteins, and of proteins involved in mitochondrial dynamics and motility (Giacomello et al., 2020). Interestingly, *Prosβ5* (and to a lesser extend *Prosβ1*; not shown) KD in flies' heart tissues led to downregulation of genes involved in mitochondria quality control system (e.g., *Pink1*/*park*) and also of the master regulator of mitochondrial biogenesis srl/PGC1‐a (Dorn 2nd et al., 2015), suggesting that proteasome KD is also likely accompanied by reduced mitophagy and/or mitochondrial biogenesis. Consistently, a number of recent studies have shown that mitochondrial perturbation greatly impacts on cardiomyocytes functionality having a crucial role in the progression of cardiovascular diseases (reviewed in, Ajoolabady et al., 2022). On the contrary, either *daw* (a TGFB‐INHB/activin‐like protein) KD or *rictor* (a TORC2 subunit) OE in fly hearts promoted cardiac autophagic flux and enhanced lifespan (Chang et al., 2020).

Notably, we also observed that heart‐targeted proteasome KD triggers systemic toxicity, as it results in developmental defects, growth retardation [indicative of defective insulin/IGF‐like signaling and metabolic deregulation (Zhang et al., 2009)], and aging acceleration; future studies will elucidate whether systemic signaling of cardiac dysfunction is mediated by specific (currently unknown) mediators in the hemolymph. Similar systemic responses in the fly model have been observed after nervous or muscle‐targeted disruption of the PN (Tsakiri, Gumeni, Iliaki, et al., 2019; Tsakiri, Gumeni, Vougas, et al., 2019) or mitostasis (Gumeni et al., 2019). Similarly, unfolded protein stress in different cellular compartments of the neuron, for example, cytoplasm (Prahlad et al., 2008), mitochondria (Durieux et al., 2011), or the ER (Taylor & Dillin, 2013) transmit the respective stress responses to distal tissues, while expression of an aggregation‐prone protein in *Caenorhabditis elegans* neurons elicits a stress signal that affects whole‐animal physiology (Berendzen et al., 2016). As mentioned, the highly integrated intracellular and/or external components of the circuit that maintains organismal proteostasis by signaling molecular perturbations across different tissues and organs remain to be identified.

Our therapeutically relevant observations indicate that damage accumulation in heart tissues due to suppression of proteasome peptidases activity can be mitigated by heart‐specific (e.g., *Atg8a/LC3* ^OE^), as well as by (systemic) dietary (e.g., PR) or pharmacological (e.g., RAP or MET) activation of autophagy. Specifically, concomitant upregulation of autophagy in heart tissues expressing low proteasome activities partially restored autophagy flux and proteome stability, upregulated heart mitochondrial number (likely via s*rl/PGC1‐a* induction) and respiratory capacity, improved cardiac activity, and also increased flies' longevity. Thus, preservation of proteome homeostasis is crucial for cardiac functionality. *Atg8a* ^OE^ in a *Prosβ5* ^RNAi^ (or *Rpt6* ^RNAi^) background (Tsakiri, Gumeni, Vougas, et al., 2019) has also been found to alleviate the toxic effects of proteasome deregulation in both muscle tissue and whole body, indicating a generalized rather than a tissue‐specific effect. Supportively, transgenic expression of *Atg8a* in the fly brain enhanced autophagy in neurons, extended flies' longevity, and increased resistance to oxidative stress (Simonsen et al., 2008), while modest heart‐specific OE of foxo [an autophagy inducer (Cheng, 2019)] in the fly model maintained cardiac proteostasis and was cardioprotective (Blice‐Baum et al., 2017). Furthermore, OE of GFP‐LC3B improved mitochondrial function and extended proliferation in HUVEC endothelial cells via activation of mitophagy (Mai et al., 2012), while mild enhancement of mitophagy can offer therapeutic benefits against cardiovascular disorders without damaging mitochondrial functionality and hence cardiomyocytes health (Ajoolabady et al., 2022). Also, suppression of activin signaling, a negatively regulator of cardiac autophagy, improved cardiac health during aging in *Drosophila* (Chang et al., 2020), and autophagy activation during fasting periods induced mitochondrial biogenesis through elevated expression of *srl/PGC1‐a* (Kapahi et al., 2010; Lee et al., 2008).

The autophagy inducer RAP or dietary restriction have been found to increase mitochondrial biogenesis in hearts of aged animals via *srl/PGC1‐a* upregulation (Chiao et al., 2016) and to extend longevity (Fontana & Partridge, 2015), while restricted diet delayed accelerated aging, improved neuronal function, and alleviated genomic stress in DNA repair‐deficient mice (Vermeij et al., 2016). It is assumed that restoration of mitostasis supplies cells with energy, which is particularly important for cardiomyocytes that are densely packed with mitochondria (Kubli & Gustafsson, 2014). Yet, as caloric restriction cannot be recommended for long life periods, an alternative autophagy‐activating approach would be physical exercise, which reportedly increases mitochondrial function and autophagic rates (Hayes et al., 2014; He et al., 2013).

Our finding that treatment of flies being exposed to BTZ or CFZ with MET (an FDA and EMA approved drug) partially restores proteostasis and mitostasis leading to largely normalized cardiac activity (as is evident by reduced bradycardia and arrhythmia) is of particular interest. The biguanide MET is an 5’ AMP‐activated protein kinase (AMPK) and autophagy inducer in cells (including cardiomyocytes) and is the first drug to be tested for its age‐targeting effects in a large clinical trial (TAME; targeting aging by MET) (Kulkarni et al., 2020). Our recent studies in mice showed that CFZ administration leads to increased activation of PP2A (upstream suppressor of both Akt and AMPKα) and subsequent reduction in phosphorylation of AMPKα, an effect, which is mitigated when MET is co‐administrated (Efentakis et al., 2019). Also, MET enhanced autophagy and was cardioprotective in δ‐sarcoglycan deficiency‐induced dilated cardiomyopathy (Kanamori et al., 2019). Consistently, MET was cardioprotective in a rat myocardial infarction model and in H9c2 cardiomyoblasts during oxygen–glucose deprivation injury by promoting autophagic flux through the AMPK pathway (Wu et al., 2021). Also, prolonged MET administration in mice decreased oxidative stress resulting in lower levels of chronic inflammation (Martin‐Montalvo et al., 2013); thus, adaptation of cells to prolonged MET administration is likely important for its beneficial effects on mitostasis. Treatment of diabetic mice with MET restores autophagy in cardiac tissue, reduces cardiomyocyte apoptosis, and protects against the development of diabetic cardiomyopathy (He et al., 2013; Xie et al., 2011). Interestingly, we found that MET administration concomitant to PI‐mediated KD of proteasome activities upregulated *Pink1*, *park,* and *srl/PGC1‐a* genes expression in flies' heart tissues; similarly, MET administration upregulated the *PINK1* and *PRKN* mRNAs expression in human mononuclear cells (Bhansali et al., 2020). Our on‐going studies with a suitable cardiac cell line, namely rat H9c2 cardiomyoblasts, have confirmed all findings shown herein in the fly model, including our observation that MET can partially restore proteasome inhibition‐mediated loss of proteostasis and mitostasis (unpublished data). Overall, the cardioprotective role of MET against therapeutic PIs is likely achieved by enhanced autophagy and mitostasis, as well as by restoring metabolic energy levels. Interestingly, we found that MET mildly reduced cathepsins activity. Early studies have demonstrated that MET targets hepatic mitochondria and modestly reduces ATP synthesis through inhibition of the respiratory chain complex I (Vial et al., 2019). Additional recent studies showed that low doses of MET can inhibit lysosomal v‐ATPase (Ma et al., 2022). We assume that partial inhibition of complex I and/or v‐ATPase activities by MET likely affects the acidophilic cathepsins activity due to lysosomal pH fluctuations; this notion highlights the necessity for a rigorous design of MET dosing regimens.

Taken together, our findings indicate that heart‐targeted proteasome dysfunction disrupts cardiac activity and triggers systemic toxicity via increased proteome, mitochondrial and metabolic instability. These data provide mechanistic explanations for the reported cardiotoxicity of therapeutic PIs in the clinic and highlight the critical threshold that has to be reached in order to gain the therapeutic anti‐tumor effect of the PIs on one hand and to avoid PN collapse on the other, especially in aged post‐mitotic tissues that express reduced proteasomal activities (Trougakos, 2019). Our observation that proteasome inhibition‐mediated cardiac dysfunction in the fly model can be alleviated by autophagic inducers (e.g., MET) is thus a relevant preclinical insight for mitigating the proteasome inhibition‐induced AEs and preventing therapy discontinuation.

## 4 EXPERIMENTAL PROCEDURES

### 4.1 Fly stocks and culture media

Fly stocks were maintained at ~25°C, 60% relative humidity on a 12 h light: 12 h dark cycle and were fed with standard medium. PR was performed for 7 days in young (7–10 days old) flies fed with standard medium containing 50% of the dry yeast extract (protein intake source) amount used in standard medium.

The transgenic strains UAS‐mito‐GFP (#8443), w^1118^ (#5905), Gal4^Mef2^ (#27390), UAS *Prosβ5* ^RNAi^ (#34810), and UAS GFP‐*Atg8a* (#51656) were obtained from the Bloomington Stock Center. The heart‐specific (Figure S9) Gal4^TinCΔ4^ driver was kindly donated by Prof. M. Frasch (Friedrich‐Alexander‐Universität, Germany). The UAS *ref(2)P‐*GFP and the UAS GFP‐*Lamp1* flies were a gift from Prof. G. Juhász (Eötvös Loránd University, Hungary).

### 4.2 Exposure of flies to drugs, locomotion, and longevity assays

All used drugs, that is, BTZ (Cayman Chemical, 179,324–69‐7), CFZ (Cayman Chemical, 868,540–17‐4), MET (Metformin; Merck, 317,240), or RAP (Rapamycin; Cayman Chemical, 53,123–88‐9) were added in flies culture medium. Young adult flies (7–10 days old) were treated with MET for 14 days and with RAP for 7 days. Larvae were grown in medium containing the respective drug until the 2nd‐3rd instar larval stage (Figure S7B). Doses (including duration) of drugs used for flies or larvae treatment are indicated in figure legends.

Neuromuscular activity (locomotion) and longevity assays were performed as described previously (Tsakiri, Gumeni, Iliaki, et al., 2019). For survival curves and statistical analyses, the Kaplan–Meier procedure and log‐rank (Mantel‐Cox) test were used; significance was accepted at *p* < 0.05. Statistical analyses and the number of the flies used for lifespan experiments are presented in figure legends and Table S1.

Experiments were performed in (≥20) young adult flies and (≥20) 3rd instar larvae unless otherwise indicated. For all shown experiments, equal number of female and male flies was used. Aged flies were selected based on control flies' longevity curves and previous reported protocols for studying aging in the fly model (Piper & Partridge, 2016; Tsakiri, Gumeni, Iliaki, et al., 2019).

### 4.3 RNA extraction and quantitative Real‐Time PCR (Q‐RT‐PCR) analyses

RNA extraction, conversion to cDNA and Q‐Real time‐PCR analysis was done as described (Tsakiri, Gumeni, Vougas, et al., 2019). Primers used (*Drosophila* genes) were the following:

*Pink1‐F: ACAGCTGGTCTACAACATCC, Pink1‐R: ACTGTAGGATCTCCGGACTG; park‐F: TTCTGCCGCAATTGTCTGCAGG, park‐R: GCATGCAACCGCCATCTCGCTC; ATPsynB‐F: CCCGTGGTGTGCAGAAAATC, ATPsynB‐R: AAACGCTGAATCTTGCGAGC; srl/PGC1‐a‐F: TGTGAGGTTAAAGCAGACGG, srl*/*PGC1‐a‐R: GTAACTTCTGAGCTTCCGTT; Atg8a‐F: ACGCCTTCGAGAAGCGTCGC, Atg8a‐R: CCAAATCACCGATGCGCGCC; Prosβ5‐F: GCCATCTACCATGCCACCTT, Prosβ5‐R: TTACCCAGCCGTCCTCCTTA; Lsd‐1‐F: ATCAGACCGATGGCCCACAG, Lsd‐1‐R: CTTCAGTTTGCGGGAGAAGC; Lsd‐2‐F: CCGAGCGCCTCCTTGAATAC, Lsd‐2‐R: GGAACTGGCATGTCATTTTCAGA; bmm/Atgl‐F: TTCACGCTCTATGACCAGCC, bmm/Atgl‐R: AGGATTGAAACACGGGGTCC*. The *RpL32/rp49* gene expression was used as a normalizer.

### 4.4 Preparation of tissue protein extracts, immunoblot analysis, detection of protein carbonyl groups, and measurement of reactive oxygen species (ROS)

Isolated heart (highly enriched) or whole‐body flies' tissues were homogenized on ice and processed for SDS‐PAGE and immunoblotting, as described previously (Tsakiri, Gumeni, Iliaki, et al., 2019); primary and secondary antibodies were applied for 1 h at RT. Blot quantitation was performed by scanning densitometry and ImageJ software (National Institutes of Health) (Figures S10 and S11).

Protein carbonyl groups were detected with the OxyBlot protein oxidation detection kit (Merk KGaA, s7150) as per the manufacturer's instructions. ROS were assayed, as previously described (Tsakiri, Gumeni, Vougas, et al., 2019); the emitted fluorescence was measured using the Spark® microplate reader (Tecan Trading AG) at excitation/emission wavelengths of 490/540 nm, respectively.

### 4.5 Measurement of proteasome, cathepsins peptidases activity, and BAF treatment

Measuring of proteasome peptidases or cathepsins activity in flies' tissues was done as described before (Tsakiri, Gumeni, Iliaki, et al., 2019). In either proteasome or cathepsins assays, the hydrolysis of the fluorogenic peptides was recorded using the Spark® microplate reader at excitation/emission wavelengths of 360/440 nm, respectively.

For BAF treatment, semi‐intact hearts, or intact larvae were incubated with 100 nM of BAF (Cayman Chemical, 11,038) in artificial hemolymph (108 mM Na^+^, 5 mM K^+^, 2 mM Ca^2+^, 8 mM MgCl_2_, 1 mM NaH_2_PO_4_, 4 mM NaHCO_3_, 10 mM sucrose, 5 mM trehalose, 5 mM HEPES, pH 7.1) and in Broadie and Bate's buffer (135 mM NaCl, 5 mM KCl, 4 mM MgCl_2_, 2 mM CaCl_2_, 5 mM TES, 36 mM Sucrose; pH 7.15), respectively, for 2 h at room temperature prior to downstream assays.

### 4.6 Mitochondria isolation and measurement of mitochondrial respiration

Mitochondria respiration rate was determined using a Clark‐type O_2_ electrode connected to a computer‐operated Oxygraph control unit (Hansatech Instruments), as described before (Gumeni et al., 2019). Temperature was maintained at 25°C, and the total reaction volume was 300 μl. The respiratory control ratio (RCR) was calculated as the ratio of State 3–State 4 (ST3/ST4).

### 4.7 Isolation of intact hearts from adult flies

To visualize the *Drosophila* beating heart, young flies were dissected as described (Vogler & Ocorr, 2009); the entire procedure was performed in an oxygenated, artificial hemolymph solution at RT. Recordings of heart activity were acquired using a BMS (Breukhoven microscopy systems/3 MB) digital microscope camera mounted on a BMS microscope. Measurements (heart beats recording) were normalized to a 30 sec period per sample; for representative movies see, Videos S1–S6.

### 4.8 Sample preparation for confocal laser scanning microscopy (CLSM)

Young flies were dissected, and flies' tissues were isolated in PBS; dissected larvae tissues were also used. Flies or larval tissues were fixed with 4% formaldehyde in PBS and permeabilized with 0.2% Triton X‐100. After blocking (3% FBS in PBS), samples were incubated with primary and secondary antibodies. Fat body and dissected heart tubes were stained with Bodipy 493/503 (Molecular Probes™/Thermo Fisher Scientific Inc., D3922) and LysoTracker Red DND‐99 (Molecular Probes™/Thermo Fisher Scientific Inc., L7528), respectively, as per manufacturer's instructions. DAPI (Molecular Probes™/Thermo Fisher Scientific Inc., D1306) and Rhodamine Phalloidin (Molecular Probes™/Thermo Fisher Scientific Inc., R415) staining (1 h at RT) were used for nuclei and F‐actin visualization, respectively.

Visualization of samples was done by using a Digital Eclipse C1 Nikon (Melville) CLSM equipped with 20 × 0.50 NA differential interference contrast (DIC), 60 × 1.40 NA DIC Plan Apochromat objectives, using the EZC1 acquisition and analysis software (Nikon). Z‐stacks with a step size of 1 μm were taken using identical settings. Each stack consisted of 26 plane images. Image J (National Institutes of Health) was used to determine fluorescence intensities.

### 4.9 Visualization of *Drosophila* hearts via micro‐computed tomography (μ‐CT)

*Drosophila* specimens were fixed in 4% formaldehyde and gradually dehydrated to 96% ethanol for 3 days. Subsequently, specimens were stained using 1% iodine dissolved in 96% ethanol (for the complete staining protocol see, Metscher, 2009) to increase the contrast between the soft tissues. *Drosophila* scans were performed with a SkyScan 1172 micro‐tomograph (Bruker). The scanner uses a tungsten source and is equipped with an 11 PM CCD camera (4000 × 2672 pixels). Samples were scanned at a voltage of 50 kV and a current of 198 μA without filter and a pixel size of ~2.90 μm for a half rotation of 180°. Projection images were reconstructed into cross sections using SkyScan's NRecon software (Bruker), which employs a modified Feldkamp's back‐projection algorithm. 3D analysis was performed for each scan using the software CT Analyser v.1.14.4.1 (Bruker) to calculate the thickness of the heart's conical chamber. Structure thickness calculation is based on the sphere‐fitting algorithm (Hildebrand & Rüegsegger, 1997). To study the dimensions of the studied flies' conical chamber, five different slices per sample (*n* = 7) were analyzed; measurements in x and y axes were taken using identical settings.

### 4.10 Measurement of growth and developmental effects

To study the effects of proteotoxic stress on development, 15 young, mated female flies were placed in Petri dishes containing 1.5% agar dissolved in sour cherry juice and allowed to lay eggs for 24 h. The embryos per dish were then counted and collected using PBS; 30 embryos from each population were then transferred in fresh standard medium and monitored for 14 days. Cultures were photographed at different developmental stages, and 20 larvae, pupae, and flies were weighted to record body weight. The numbers of pupae and adult flies were also measured.

### 4.11 Antibodies

The primary antibodies used were the anti‐Ubiquitin (Santa Cruz Biotechnology, Inc., sc‐8017), anti‐foxo (COSMO BIO CO, CAC‐THU‐A‐DFOXO), anti‐26S proteasome α (Santa Cruz Biotechnology, Inc., sc‐65,755), anti‐26S Proteasome p54 (Rpn10) (Santa Cruz Biotechnology, Inc., sc‐65,746), anti‐pAMPK (Cell Signaling Technology, #2535), anti‐GABARAP (Atg8a) (Cell Signaling Technology, #13733), anti‐Lamp1 (Abcam, ab30687), anti‐ATP5A (Abcam, ab14748), anti‐Actin (Cell Signaling Technology, #8457), and anti‐Gapdh (Sigma‐Aldrich, G9545). The anti‐ref(2)P/p62 antibody was kindly donated from Prof. G. Juhász. The secondary antibodies Peroxidase AffiniPure Donkey anti‐Mouse IgG (715‐035‐150) and Peroxidase AffiniPure Donkey anti‐Rabbit IgG (711‐035‐152) were purchased from Jackson ImmunoResearch Laboratories, Inc. The anti‐Rabbit‐IgG Alexa Fluor 647 (711‐605‐152), anti‐Rabbit‐IgG Alexa Fluor 488 (111‐545‐003), anti‐Mouse‐IgG Rhodamine (TRITC) AffiniPure (715‐025‐151), anti‐Mouse‐IgG Alexa Fluor 488 (115‐545‐003), and anti‐Mouse IgG DyLight™ 405 (715‐475‐151) antibodies were also from Jackson ImmunoResearch Laboratories, Inc. Ponceau S solution (6226–79) was from Sigma‐Aldrich.

### 4.12 Quantitation and statistical analyses

Experiments were performed at least in triplicates (for each biological replicate, *n* ≥ 3; unless otherwise indicated). For statistical analysis, the GraphPad Prism 8.0, the MS Excel, and the Statistical Package for Social Sciences (IBM SPSS; version 25.0 for Windows) were used. Statistical significance was evaluated using unpaired *t* test or one‐way ANOVA test followed by Kruskal–Wallis test (see, figure legends). Data points correspond to the mean of the independent experiments and error bars denote standard deviation (SD); significance at *p < 0.05* or *p < 0.01* is indicated in graphs by one or two asterisks, respectively. For flies' survival curves and statistical analyses, the Kaplan–Meier procedure and log‐rank (Mantel‐Cox) test were used (significance was accepted at *p* < 0.05). Statistics for the longevity experiments are reported in figure legends and in Table S1.

## AUTHOR CONTRIBUTIONS

IPT designed and supervised the study; EDP, SG, ADS, AR, and IPT conducted experiments or interpreted the data; KK performed the μ‐CT‐Scan analyses; ET, EK, KS, GPS, and MAD generated or contributed reagents, materials, or analysis tools; EDP and IPT wrote the manuscript. All authors discussed the results and commented on the manuscript.

## CONFLICT OF INTEREST

None declared.

## Supporting information


Appendix S1

Video S1

Video S2

Video S3

Video S4

Video S5

Video S6

## Data Availability

The datasets generated and/or analyzed during the current study are available from the corresponding author on reasonable request.
