# Supplementary material for: Autophagy activation can partially rescue proteasome dysfunction‐mediated cardiac toxicity
Source: Aging Cell. 2022 Oct 19;21(11):e13715. doi: 10.1111/acel.13715 (PMC9649605; doi:10.1111/acel.13715)
Supplement: Supplementary file 1 — Appendix S1 [file ACEL-21-e13715-s001.pdf]

Autophagy activation can partially rescue proteasome dysfunction-mediated cardiac toxicity

Eleni-Dimitra Papanagnou, Sentiljana Gumeni, Aimilia D. Sklirou, Alexandra Rafeletou, Evangelos Terpos, Kleoniki Keklikoglou, Efstathios Kastritis, Kimon Stamatelopoulos, Gerasimos P. Sykiotis, Meletios A. Dimopoulos, Ioannis P. Trougakos

SUPPORTING INFORMATION

SUPPORTING FIGURE LEGENDS

Figure S1 **Aging related phenotypes in flies' cardiac tissue.** (A) Relative (%) CT-L proteasome activity in aged *vs*. young flies. (B) CLSM visualization (B1) and immunoblotting analyses (B2) of isolated heart tubes from young and aged w^{1118} flies probed with an anti-ref(2)P/p62 antibody; nuclei were counterstained with DAPI. (C) CLSM viewing after staining with LysoTracker (C1), LysoTracker quantitation (C2) and immunoblotting analyses using the lysosomal marker anti-Lamp1 (C3) in young and aged w^{1118} flies' heart tubes. (D) Relative (%) cathepsins activity in heart tissues of flies aged *vs*. young flies. (E) Heart beats (mean from 10 female adult flies) normalized to a 30 sec period. (F) Heart rhythm calculated by beats per 5 seconds; each dot represents the number of heart beats/5 seconds. Actin probing in (B2), (C3) was used as protein input reference. *P* values were calculated with unpaired t-Test. Bars, ± SD (n ≥ 3); *P < 0.05; **P < 0.01.

Figure S2 Increased Atg8a-proteasome co-localization after heart-targeted *Prosβ5* KD. (A) Relative (%) 26S proteasome activities (*vs*. control) in various tissues of *Prosβ5*, Gal4$^{TinC\Delta4}$ transgenic flies. (B) Representative immunofluorescence images of isolated heart tubes from *Prosβ5*, Gal4$^{TinC\Delta4}$ flies co-stained for Atg8a/GABARAP and 26S-α or P54/Rpn10 proteasomal subunits (nuclei were counterstained with DAPI) (B1) along with immunoblotting analyses using anti-26S-α or anti-P54/Rpn10 antibodies (B2). Ponceau S staining was used as total protein input reference, respectively. *P* values were calculated with unpaired t-Test. Bars ± SD (n = 3); $*P < 0.05$.

Figure S3 Prolonged *Prosβ5* KD suppresses autophagic flux leading to disrupted lysosomal acidification. (A) Immunoblotting analyses of protein samples from heart dissected tissues of *Prosβ*5 KD flies treated (or not) with bafilomycin A1 (BAF); samples were probed with antibodies against Atg8a/GABARAP. Asterisks (*) indicate the lipidated Atg8a form. (B) CLSM visualization of ref(2)P-GFP along with Atg8a/GABARAP immunofluorescence staining in larval muscle tissue following muscle-targeted (Gal4^{Mef2}) *Prosβ5* KD; nuclei were counterstained with DAPI. Arrows indicate the nuclear localization of Atg8a/GABARAP. (C) Immunoblot analyses showing ref(2)P-GFP and ref(2)P/p62 (endogenous) expression levels (*vs*. control) in larval tissues of a *Prosβ5*^{RNAi}, *ref(2)P*-GFP/Gal4^{Mef2} transgenic line. (D) CLSM visualization of GFP-*Lamp1* and LysoTracker staining in control or after muscle-targeted *Prosβ5* KD larval heart tissue (Gal4^{Mef2}). Actin probing (A) and Ponceau S staining (C) were used as protein input reference. Experiments in (B-D) were

performed in viable 2nd-3rd instar larvae since muscle targeted *Prosβ5* KD in *Drosophila* induces lethality at 3rd instar larval stage and pupae (Tsakiri, Gumeni, Vougas, et al., 2019). For shown experiments, (n = 3).

Figure S4 ***Atg8a* OE in the *Prosβ5* KD background increases autophagic flux in the heart and partially rescues proteasome KD-mediated developmental defects, growth retardation and disrupted structure of heart tube in the adult.** (A) Relative expression levels of *Prosβ5* and *Atg8a* genes in young flies of the shown genotypes. (B) Immunoblotting analyses of protein samples from semi-intact heart (B1) and whole body (B2) tissues of the shown transgenic flies treated (or not) with BAF; samples were probed with antibody against Atg8a/GABARAP, as indicated. (C) Immunoblot analyses of proteome ubiquitination (Ub) and carbonylation (DNP) in flies' heart tissues of the indicated genotypes. (D) Representative images (D1) and weight (%) (D2) of larvae, pupae, and female adult young flies of the shown genotypes and number (%) (D3) of pupae and hatched flies at 7- and 14-days, respectively post transferring 30 embryos per assay to culture medium. (E) Representative μ-CT images of the heart tube (E1) along with thickness quantitation (left panel) and measurement of the dimensions in x and y axes (right panel) (E2) of the heart at the indicated transgenic flies; analyses were performed in the conical chamber of the tube. Gene expression in (A) was plotted *vs*. the respective control; *RpL32/rp49* was used as reference. Asterisks (*) in (B) indicate the lipidated Atg8a form. In (B, C) Actin and Gapdh probing were used as protein input reference respectively. *P* values were calculated with one-way ANOVA with Kruskal Wallis test. Bars, ± SD (n = 3); *P < 0.05; **P < 0 01.

Figure S5 Concomitant PR in flies with heart-specific proteasome KD improves cardiac functionality and longevity. (A) Representative immunofluorescence images of isolated heart tubes from +/GFP-*Lamp1*, Gal4$^{TinC\Delta4}$ flies stained for Atg8a/GABARAP after 7 days exposure to PR medium (A1) and immunoblot analyses of adult flies' somatic tissues protein samples probed with antibodies against p-Ampka and foxo proteins (A2). (B) CLSM visualization following immunofluorescence staining with blw/ATP5A of the shown transgenic flies' heart tissues. (C, D) Heart rhythm of *Prosβ5*^{RNAi} (C1) flies exposed (or not) to PR medium calculated by beats per 5 seconds and heart beats (mean from 15 female adult flies) normalized to a 30 sec period (C2) (see, Videos S2, S4); each dot in (C1) represents the number of heart beats/5 seconds. (D) Longevity curves of flies with the shown genotype treated with standard or PR medium; Log-rank, Mantel-Cox test: *Prosβ5*^{RNAi} *vs.* *Prosβ5*^{RNAi}-PR, P=0.03. Statistics of the longevity curves are also reported in Table S1. Actin probing and Ponceau S staining in (A) were used as protein input reference. *P* values were calculated with unpaired t-Test. Bars, ± SD (n ≥ 3); *P < 0.05; **P < 0.01.

Figure S6 Administration of RAP to PIs-treated young flies preserves cardiac and mitochondria function. (A) Relative (%) 26S proteasome activities in w^{1118} flies heart tissues after treatment (or not) with the indicated drugs for 7 days. (B) CLSM visualization of mitochondria after immunofluorescence staining of blw/ATP5A in w^{1118} flies dissected heart tissues. (C) Heart beats (mean from 15 female adult flies) normalized to a 30 sec period. (D) Heart rhythm calculated by beats per 5 seconds; each dot represents the number of heart beats/5 seconds. Drugs' concentrations

used in flies were as follows: RAP (100 μM), CFZ (50 μM), BTZ (1 μM). *P* values in (A, C) were calculated with one-way ANOVA with Kruskal Wallis test and in (D) with unpaired t-Test. Bars, ± SD ($n \geq 3$); *$P < 0.05$; **$P < 0.01$.

Figure S7 **MET administration increases autophagy flux in flies' cardiac tissue and reduces the PIs-mediated ref(2)P/p62 accumulation in the muscle of both larvae and adult flies.** (A) Representative immunofluorescence images of isolated heart tubes from +/GFP-*Lamp1*, $Gal4^{TinC\Delta4}$ transgenic flies stained for Atg8a/GABARAP; mitochondria were stained with blw/ATP5A. (B) CLSM viewing of *ref(2)P*-GFP/$Gal4^{Mef2}$ larval muscles after treatment with the respective drugs; samples were co-stained with anti-Atg8a/GABARAP. Arrows indicate the nuclear localization of Atg8a/GABARAP. (C) Representative immunofluorescence images of *ref(2)P*-GFP/$Gal4^{Mef2}$ adult transgenic flies' muscles following treatment with the indicated drugs. Rectangles in (A) denote areas shown in high magnification; arrows indicate sites of Lamp1 and Atg8a co-localization. DAPI in (B) was used to counterstain nuclei. Young flies were treated with MET for 14 days. Used drugs concentrations were as follows: MET (1 mM), BTZ (1 μM), CFZ (50 μM); treatment of larvae with CFZ was lethal. (n = 3).

Figure S8 **MET administration reduces lipid droplets in flies' fat body and increases expression of the lipolysis related *Atgl/bmm* gene.** (A) CLSM representative images of adipose tissue in flies following treatment with the shown drugs (A1) and quantitation of the total lipid droplets (LD) number (A2). (B) Relative expression levels of shown lipolysis and lipogenesis-related genes in heart tissues of flies treated

with the indicated drugs. Gene expression in (B) was plotted *vs*. the respective control; *RpL32/rp49* was used as reference. The concentration of used drugs was as follows: MET (1 mM), BTZ (1 µM), CFZ (50 µM). *P* values were calculated with one-way ANOVA with Kruskal Wallis. Bars ± SD (n ≥ 3); *P < 0.05; **P < 0.01.

Figure S9 **Gal4$^{TinC\Delta4}$ driver tissue-specificity.** Confocal images of adult flies' heart, gut, neuronal tissue and ovaries after heart tissue specific expression (Gal4$^{TinC\Delta4}$) of mito-GFP reporter.

Figures S10-S11 **Quantitation of immunoblots.** Relative expression of the studied proteins *vs*. the respective protein input reference.

SUPPORTING TABLE

Table S1 Summary of lifespan experiments.

SUPPORTING VIDEOS

Representative videos of heart beats in young female insects per genotype and/or treatment.

Video S1 +/Gal4$^{TinC\Delta4}$

Video S2 *Prosβ5*^{RNAi}/Gal4$^{TinC\Delta4}$

Video S3 *Prosβ5*^{RNAi}, GFP-*Atg8a*^{OE}/Gal4$^{TinC\Delta4}$

Video S4 *Prosβ5*^{RNAi}/Gal4$^{TinC\Delta4}$ - PR treatment

Video S5 BTZ 1 µM treatment

Video S6 BTZ 1 µM + MET 1 mM treatment

SUPPORTING LIST OF GENES ASSAYED IN *Drosophila melanogaster*

Atg8a (Autophagy-related 8a, FBgn0052672, CG32672); *Atgl/bmm* (brummer, FBgn0036449, CG5295); *ATPsynB* (ATP synthase, subunit B, FBgn0019644, CG8189); *Lsd-1* (Lipid storage droplet-1, FBgn0039114, CG9057); *Lsd-2* (Lipid storage droplet-2 FBgn0030608, CG10374); *park* (parkin, FBgn0041100, CG10523); *Pink1* (PTEN-induced putative kinase 1, FBgn0029891, CG4523); *Prosβ5* (Proteasome β5 subunit, FBgn0029134, CG12323); *RpL32/rp49* (Ribosomal protein L32, FBgn0002626, CG7939); *srl/PGC1-a* (spargel, FBgn0037248, CG9809).

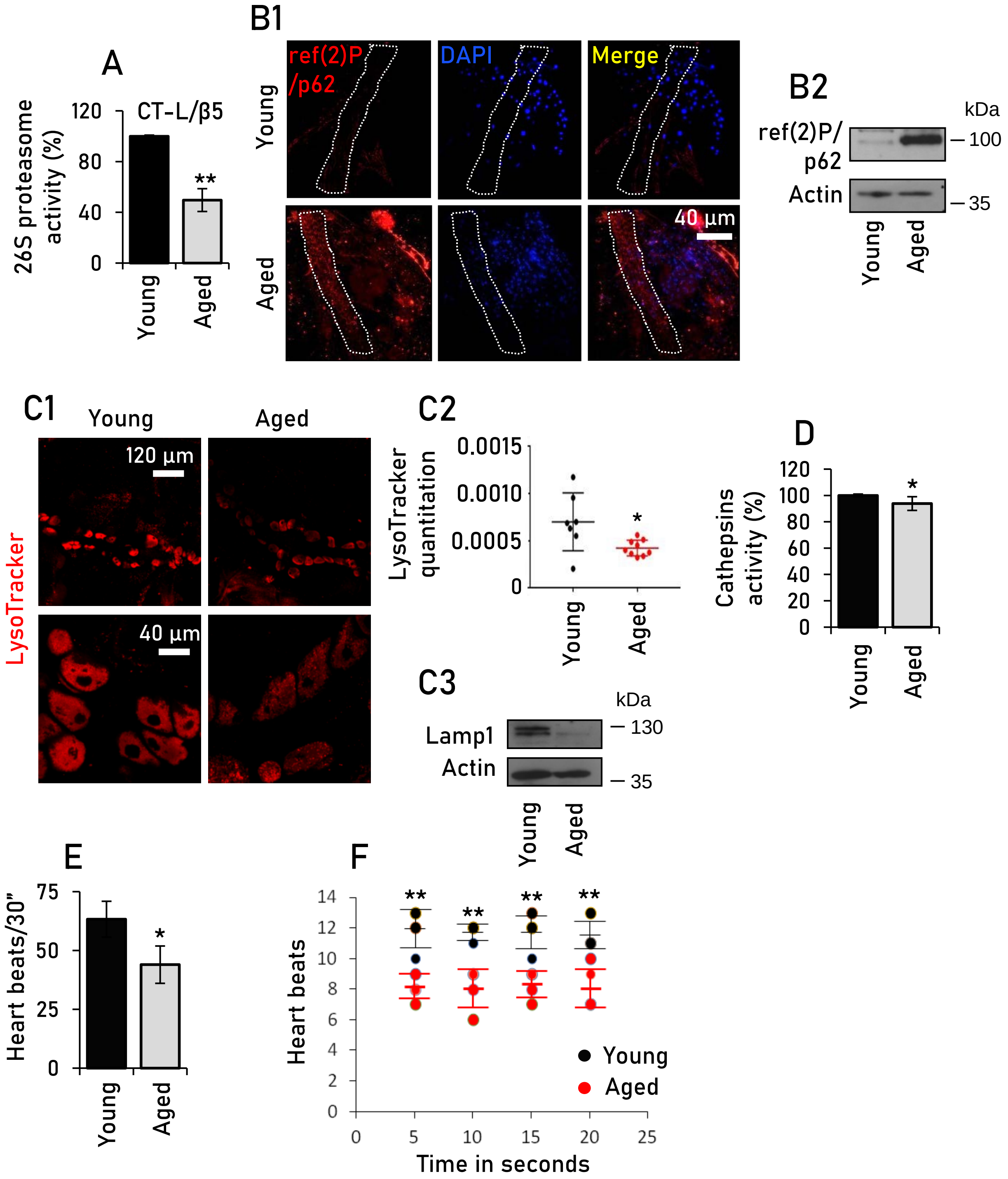

Papanagnou et al. Figure S1

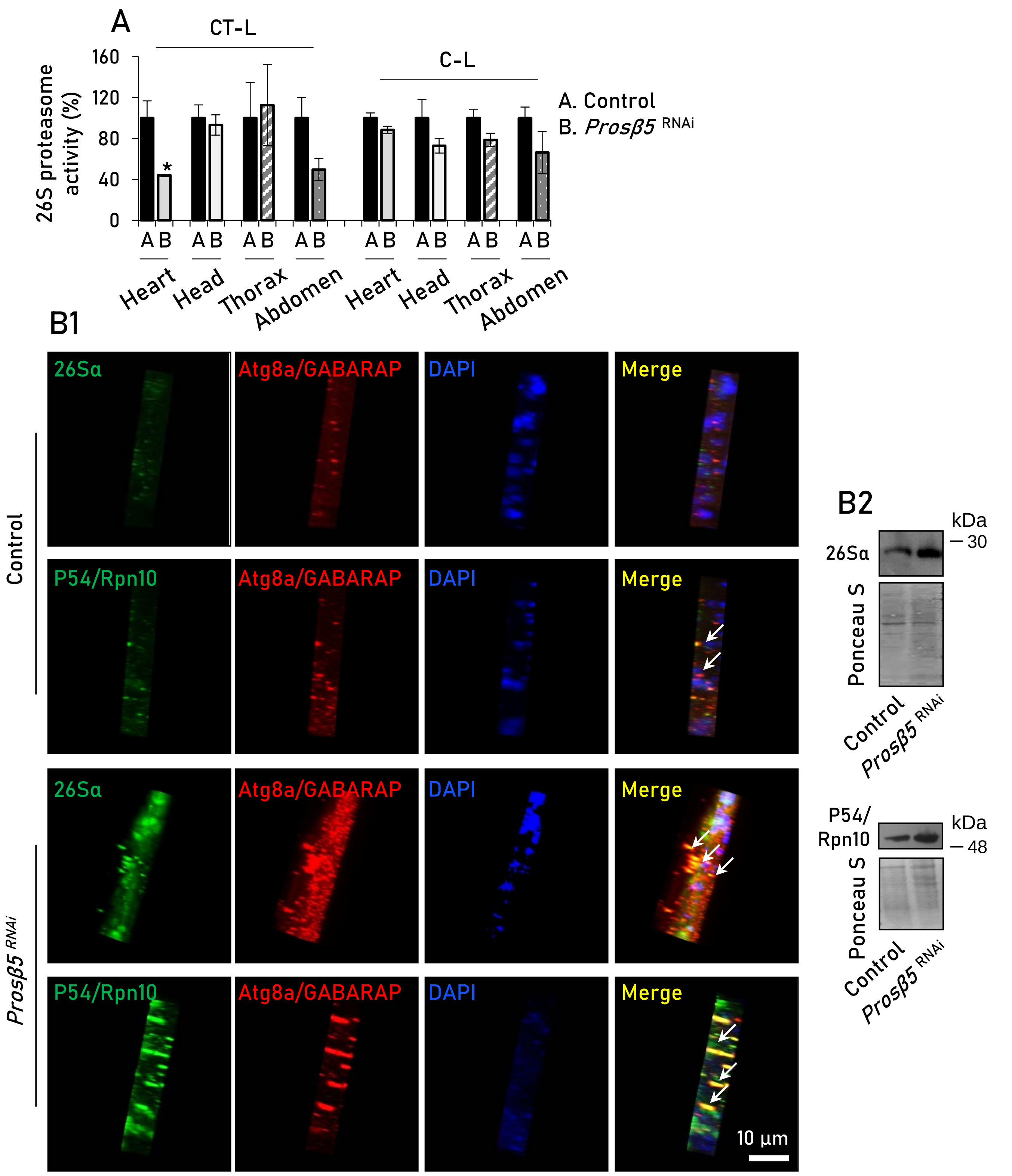

Papanagnou et al. Figure S2

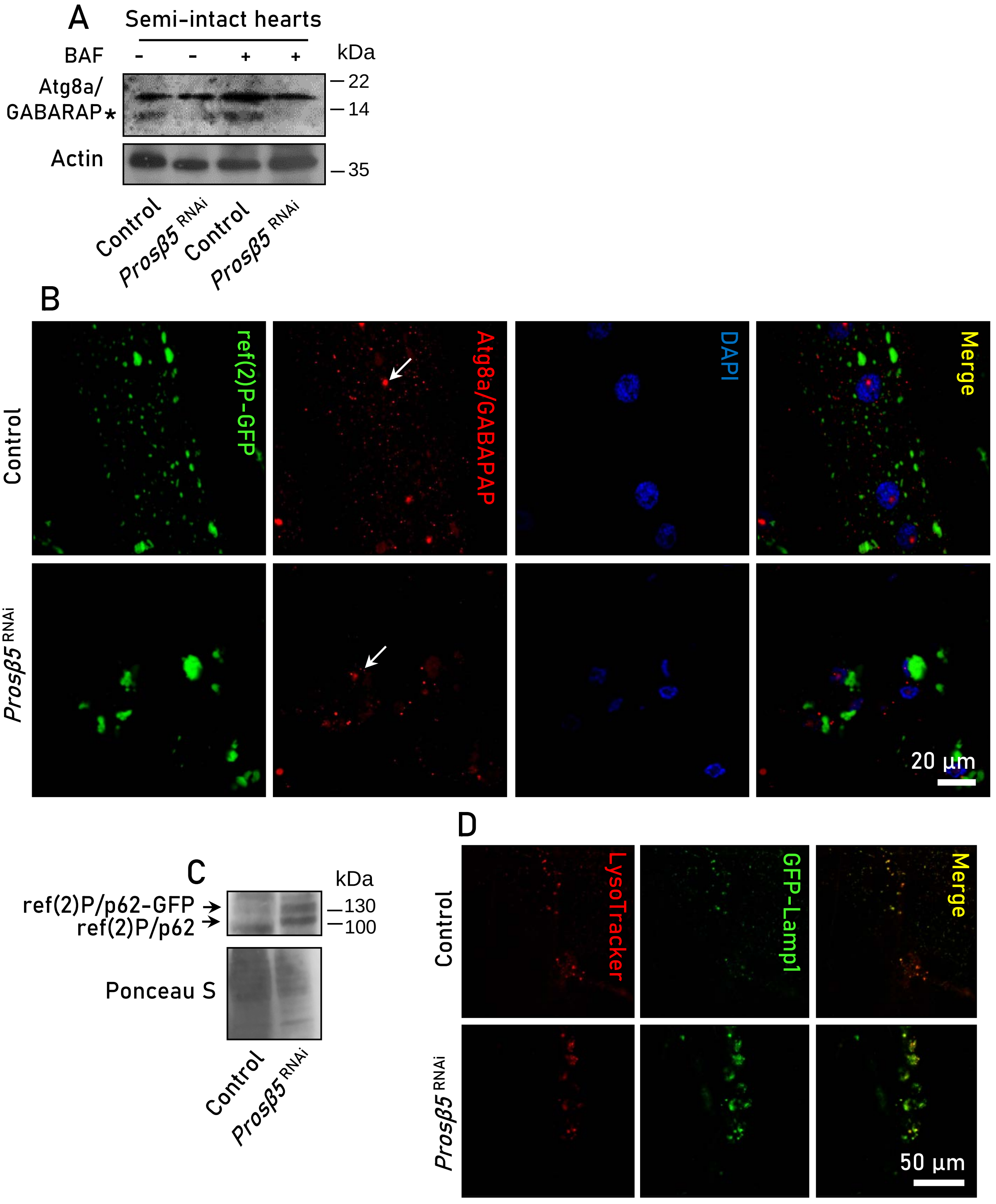

Papanagnou et al. Figure S3

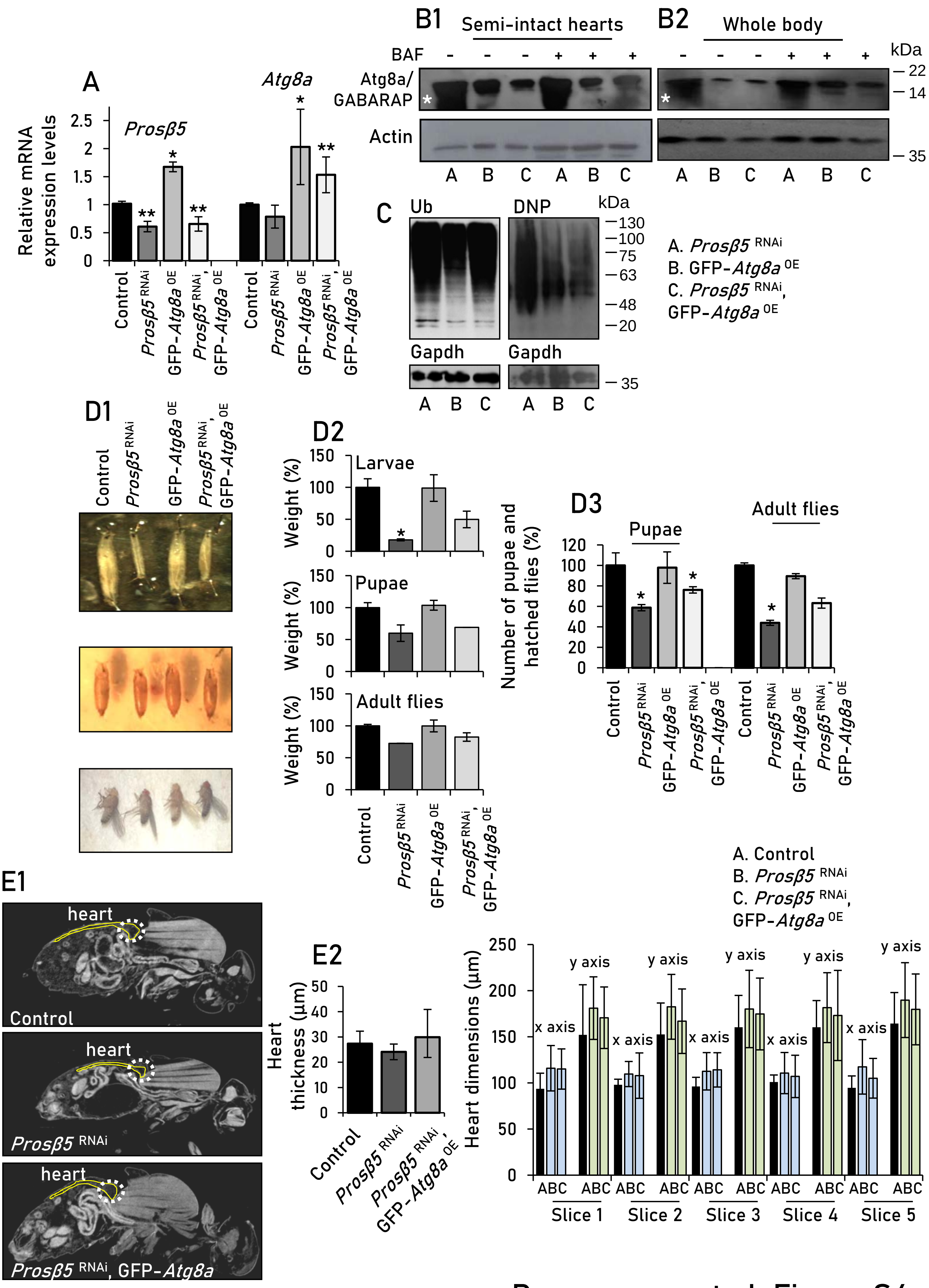

Papanagnou et al. Figure S4

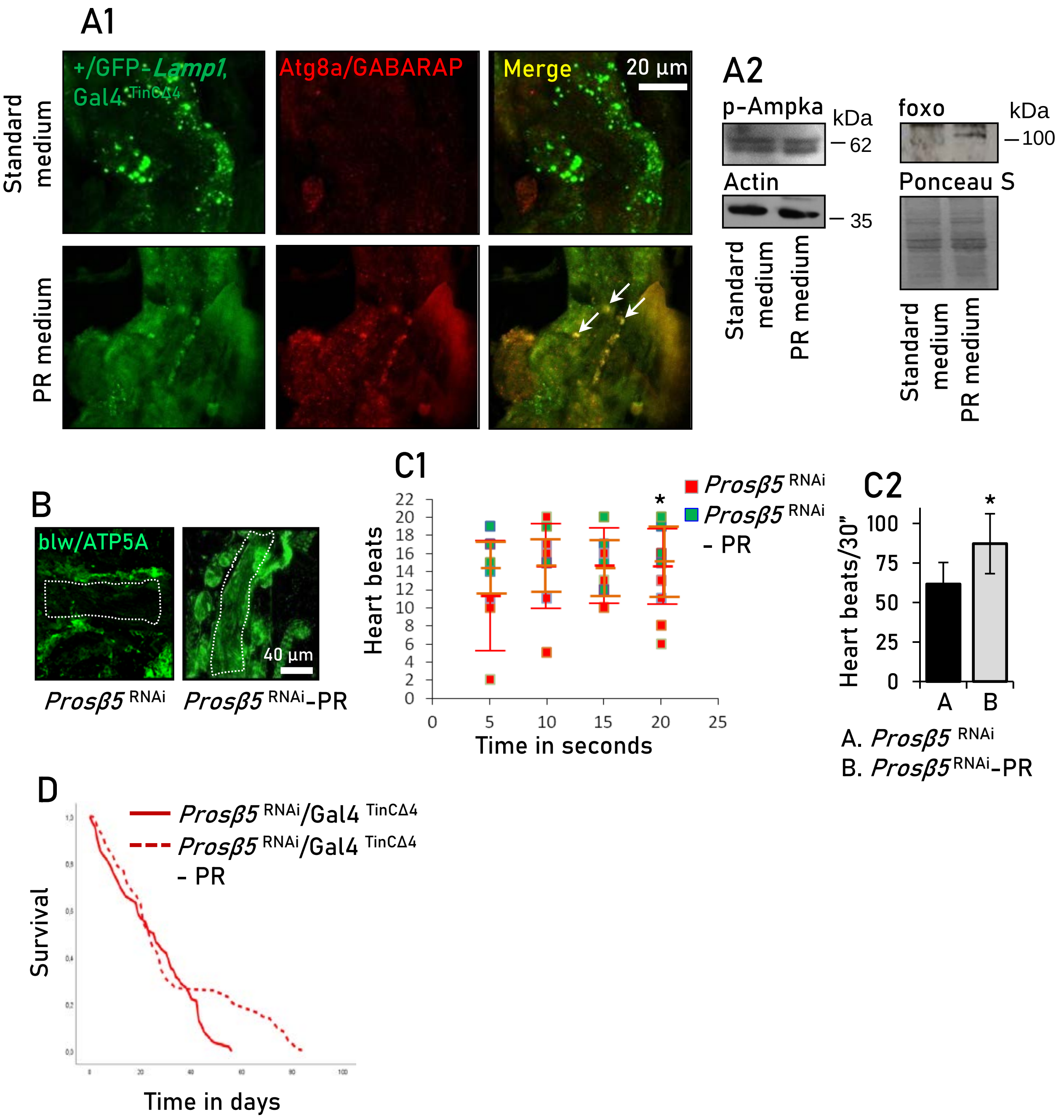

Papanagnou et al. Figure S5

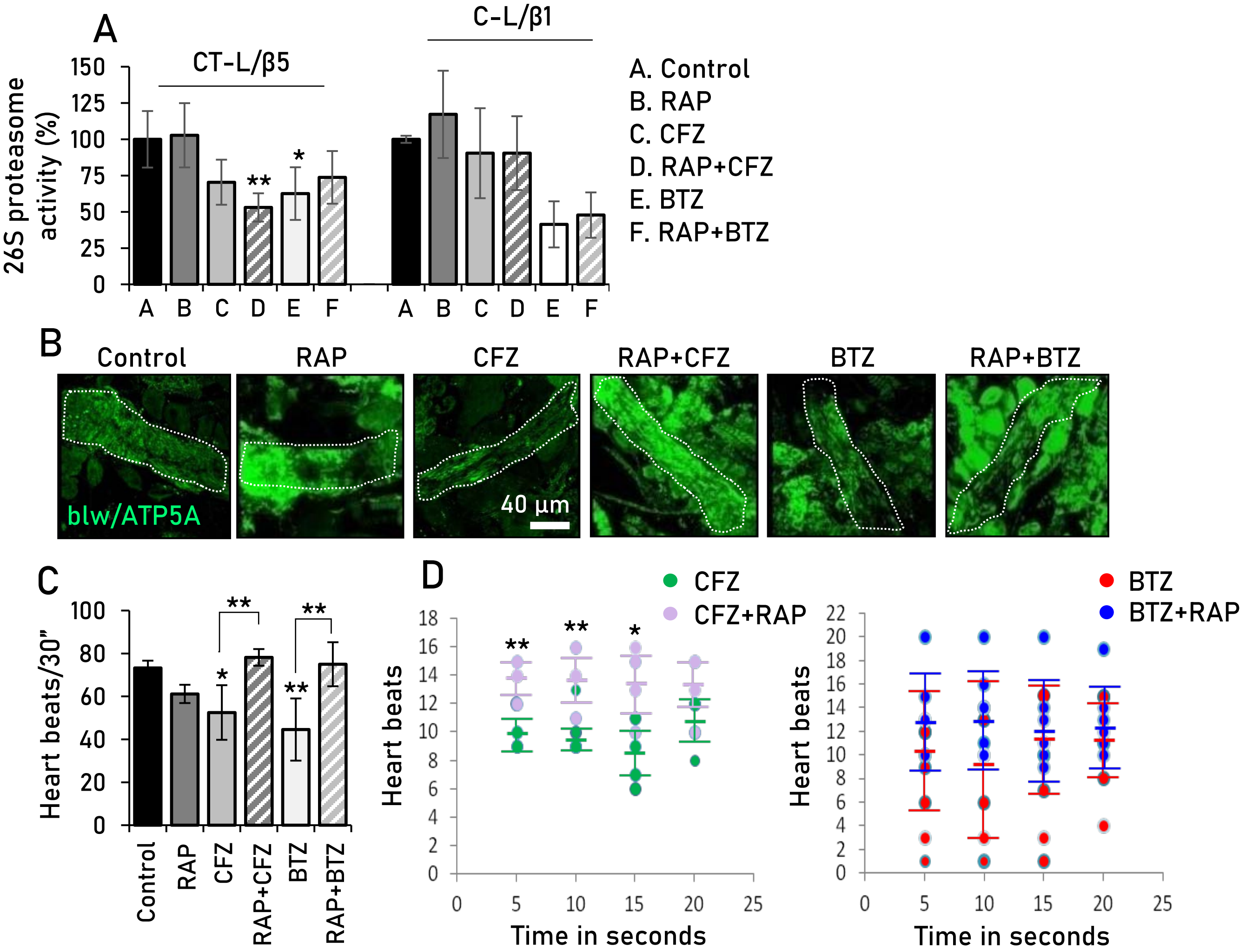

Papanagnou et al. Figure S6

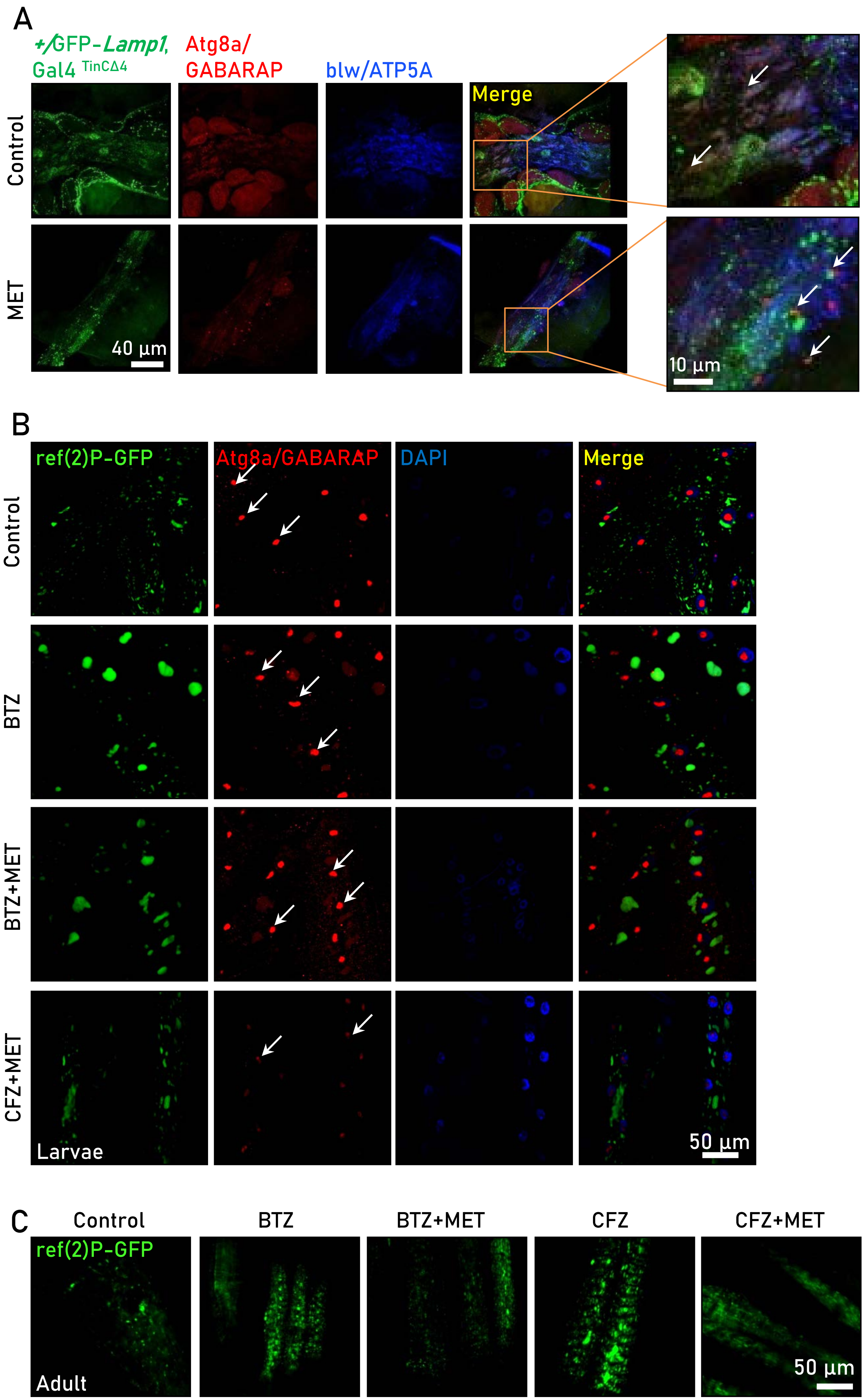

Papanagnou et al. Figure S7

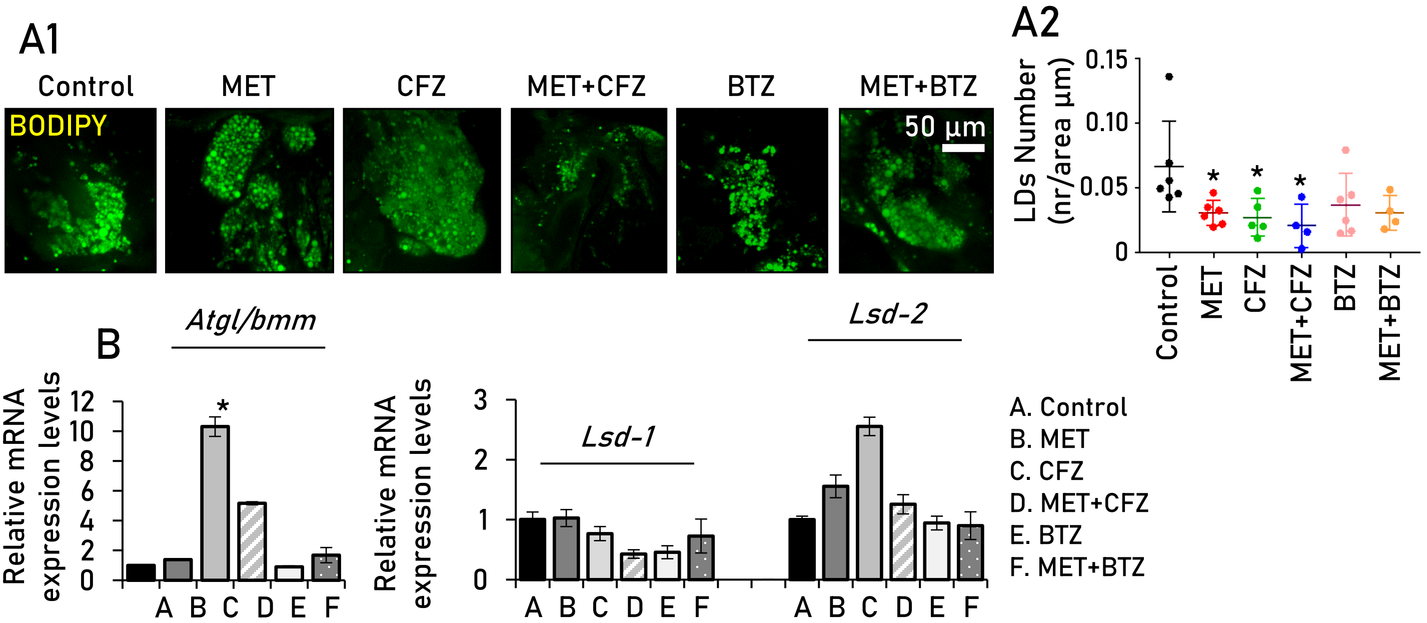

Papanagnou et al. Figure S8

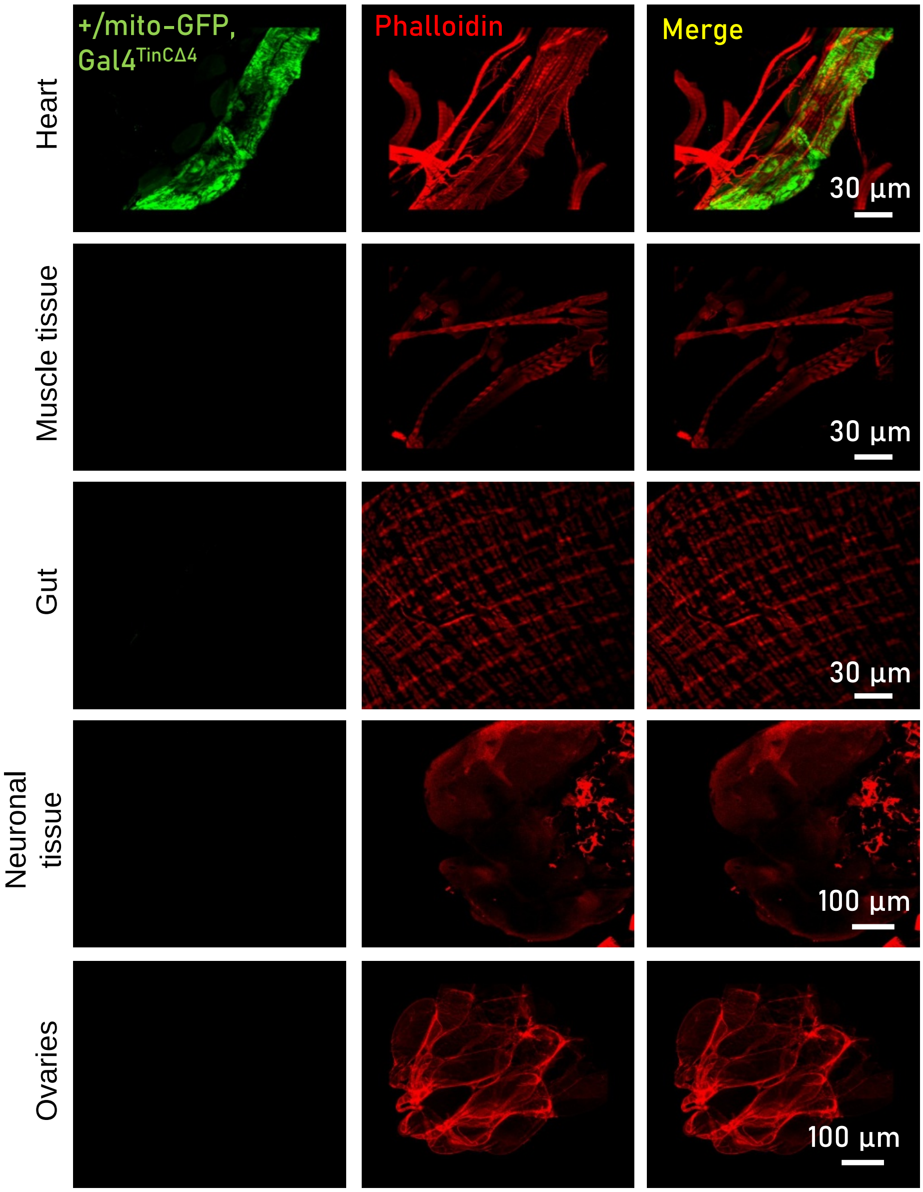

Papanagnou et al. Figure S9

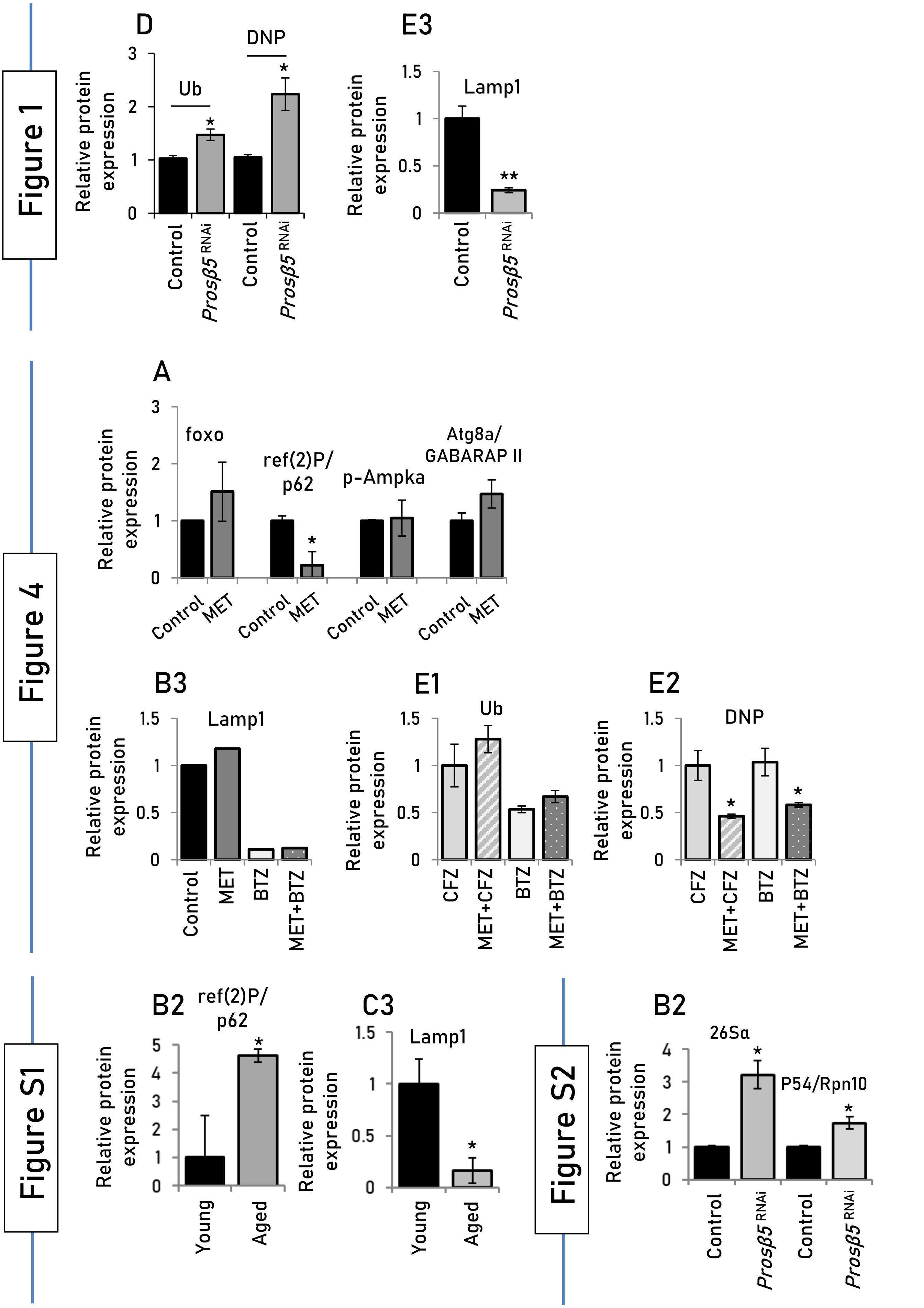

Papanagnou et al. Figure S10

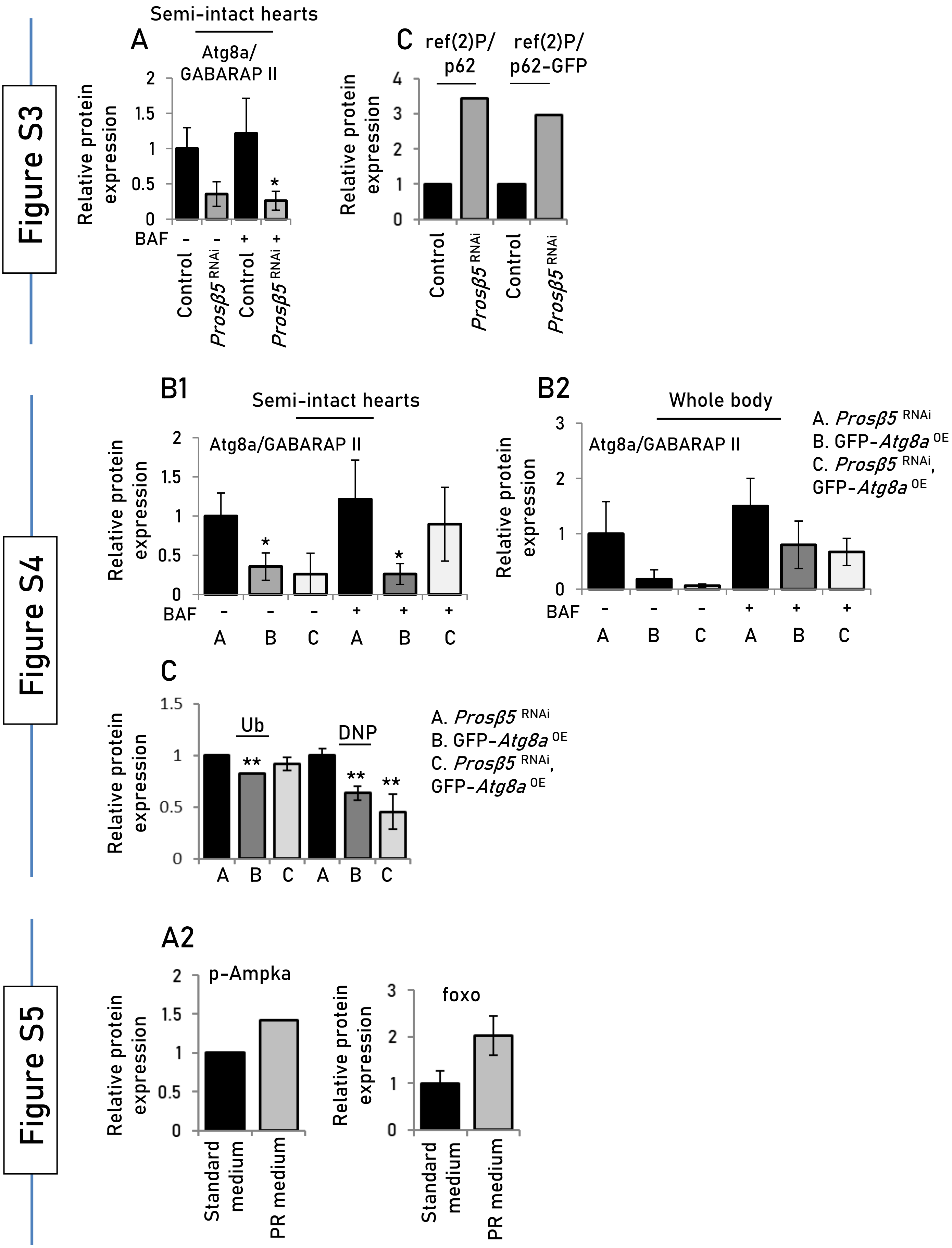

Papanagnou et al. Figure S11

Table S1. Summary of lifespan experiments.

Figure	Sample	Mean Lifespan (L.F) +/- s.e.m. (Days)		Median L.F +/- s.e.m. (Days)		% Median LF *vs*. control	Max (Days)	Log Rank P Value						
								+/Gal4$^{TinC\Delta4}$	UAS *Prosβ5*^{RNAi}/Gal4$^{TinC\Delta4}$					*Total Animals Died/Total*
Figure 2G	+/Gal4$^{TinC\Delta4}$	62	2.03	71	3.02	100	98		0					158/158
	UAS *Prosβ5*^{RNAi}/Gal4$^{TinC\Delta4}$ (*)	30	1.08	32	1.57	45	56	0						149/158
								UAS *Prosβ5*^{RNAi}/Gal4$^{TinC\Delta4}$	UAS *Prosβ5*^{RNAi}, UAS GFP-*Atg8a*/Gal4$^{TinC\Delta4}$					*Total Animals Died/Total*
Figure 3K	UAS *Prosβ5*^{RNAi}/Gal4$^{TinC\Delta4}$ (*)	30	1.08	32	1.57	45	56		0					149/158
	UAS *Prosβ5*^{RNAi}, UAS GFP-*Atg8a*/Gal4$^{TinC\Delta4}$	37	1.44	44	1.89	62	68	0						155/158
								Control	MET	CFZ	CFZ+MET	BTZ	BTZ+MET	*Total Animals Died/Total*
Figure 5G	Control	36	1.55	34	2.66	100	73		0.5	0	0	0	0	115/120
	MET	36	1.76	37	3.79	109	66	0.5		0	0	0	0	115/120
	CFZ	17	0.64	16	1.14	47	30	0	0		0.03	0.88	0	120/120
	CFZ+MET	18	0.78	18	0.97	53	33	0	0	0.03		0.08	0	120/120
	BTZ	17	0.64	15	0.50	44	33	0	0	0.88	0.08		0	120/120
	BTZ +MET	23	0.91	23	1.56	68	38	0	0	0	0	0		120/120
								UAS *Prosβ5*^{RNAi}/Gal4$^{TinC\Delta4}$	UAS *Prosβ5*^{RNAi}/Gal4$^{TinC\Delta4}$ - PR					*Total Animals Died/Total*
Figure S5D	UAS *Prosβ5*^{RNAi}/Gal4$^{TinC\Delta4}$	24	1.55	23	2.67	100	56		0.03					100/102
	UAS *Prosβ5*^{RNAi}/Gal4$^{TinC\Delta4}$- PR	32	2.45	24	1.31	104	85	0.03						101/103
